# Health economic evaluation of moist wound care in chronic cutaneous leishmaniasis ulcers in Afghanistan

**DOI:** 10.1186/s40249-018-0389-4

**Published:** 2018-02-14

**Authors:** Hans-Christian Stahl, Faridullah Ahmadi, Sami Mohammad Nahzat, Heng-Jin Dong, Kurt-Wilhelm Stahl, Rainer Sauerborn

**Affiliations:** 1 Institute of Public Health, University Hospital Heidelberg, Heidelberg, Germany; 2 Malaria and Leishmaniasis Centre, Provincial Civil Balkh Hospital, Mazar-e-Sharif, Afghanistan; 3 National Malaria and Leishmaniasis Control Program, Ministry of Public Health, Kabul, Afghanistan; 4 Center for Health Policy Studies, School of Medicine, Zhejiang University, Hangzhou, China; 5 NGO Waisenmedizin e.V. – Promoting Access to Essential Medicine, Freiburg, Germany

**Keywords:** Cutaneous leishmaniasis, Wound healing, Cost-effectiveness, Sodium Stibogluconate, Moist wound treatment

## Abstract

**Background:**

The present health economic evaluation in Afghanistan aims to support public health decision makers and health care managers to allocate resources efficiently to appropriate treatments for cutaneous leishmaniasis (CL) elicited by *Leishmania tropica or Leishmania major*.

**Methods:**

A decision tree was used to analyse the cost and the effectiveness of two wound care regimens versus intra-lesional antimony in CL patients in Afghanistan. Costs were collected from a societal perspective. Effectiveness was measured in wound free days. The incremental cost-effectiveness ratio (ICER) and incremental net monetary benefit (NMB) were calculated. The model was parameterized with baseline parameters, sensitivity ranges, and parameter distributions. Finally, the model was simulated and results were evaluated with deterministic and probability sensitivity analyses. Final outcomes were the efficiency of the regimens and a budget impact analysis in the context of Afghanistan.

**Results:**

Average costs per patients were US$ 11 (*SE* = 0.016) (Group I: Intra-dermal Sodium Stibogluconate [IL SSG]), US$ 16 (*SE* = 7.58) (Group II: Electro-thermo-debridement [ETD] + Moist wound treatment [MWT]) and US$ 25 (*SE* = 0.48) (Group III: MWT) in patients with a single chronic CL ulcer. From a societal perspective the budget impact analysis shows that the regimens’ drug costs are lower than indirect disease cost. Average effectiveness in wound free days are 177 (*SE* = 0.36) in Group II, 147 (*SE* = 0.33) in Group III, and 129 (*SE* = 0.27) in Group I. The ICER of Group II versus Group I was US$ 0.09 and Group III versus Group I US$ 0.77, which is very cost-effective with a willingness-to-pay threshold of US$ 2 per wound free day. Within a Monte-Carlo probabilistic sensitivity analysis Group II was cost-effective in 80% of the cases starting at a willingness-to-pay of 80 cent per wound free day.

**Conclusions:**

Group II provided the most cost-effective treatment. The non-treatment alternative is not an option in the management of chronic CL ulcers. MWT of Group III should at least be practiced. The cost-effectiveness of Group III depends on the number of dressings necessary until complete wound closure.

**Electronic supplementary material:**

The online version of this article (doi: 10.1186/s40249-018-0389-4) contains supplementary material, which is available to authorized users.

## What is already known about the topic?

For nearly a century Sodium Stibogluconate (SSG) has worldwide been the mainstay treatment in cutaneous leishmaniasis (CL), especially in Afghanistan. CL chronic lesions are assumed to be self-healing [1, 2]. Therefore, the World Health Organization (WHO) CL case management guidelines rightly promote simple aseptic moist wound care in the first place and subsequently intra-dermal Sodium Stibogluconate (IL SSG) injections, in case CL lesions become chronic [3, 4].

## What does this paper add to existing knowledge?

To our knowledge the paper is the first published health economic investigation on wound care in chronic CL ulcers. The analysis shows that simple wound debridement and moist wound treatment is an efficient treatment alternative to intra-dermal sodium stibogluconate in CL ulcers in 80% of the cases. Results are based on direct medical, direct non-medical, indirect costs and efficacy data collected within a published randomized clinical trial. Direct non-medical cost and indirect costs have the most important share in total average patient costs. Indirect and direct non-medical costs have so far been neglected in the health economic analysis of wound care in chronic wounds. Decreasing treatment compliance and/or efficacy due to parasitic resistance negatively affect the comparative efficacy of the standard IL SSG regimen compared to MWT. The non-treatment alternative is not an option in the management of chronic CL ulcers, due to the indirect cost.

## (Optional) What insights does this paper provide for informing health care-related decision making?

The health economic analyses of the clinical results show that the proposed MWT is a cost-effective treatment in CL ulcers with or without prior wound debridement using high frequency electro-thermo-debridement (HF ETD). Methodologically, health economic research in CL should systematically consider quantifying indirect cost, the cost-of-illness associated with the disease and additionally account for patients’ heterogeneity. Models should be adapted to the regional health care settings so as to support national health care decision-making.

## Remark from the first-author

The magistral DAC N-055 formulation as contained in the German Drug Codex (DAC) dated of the year 2014 can be requested from the corresponding author, as well as the original TreeAge file with the decision analytical health economic model and the ethical clearances and approvals.

## Multilingual abstract

Please see Additional file 1 for translations of the abstract into the five official working languages of the United Nations.

## Background

There is currently no vaccine available to prevent from scars and disfirgurement due to Old World Cutaneous Leishmaniasis (OWCL). Although not lethal, cutaneous leishmaniasis (CL) is treated in order to reduce infections and increase hygiene in crusted ulcers with bio-films, to kill the parasite, to reduce scarring, especially in the face, by accelerating wound healing and to prevent relapses.

Since the discovery of the less toxic pentavalent antimonials in the late 1920s that led to the synthesis of sodium stibogluconate in 1945 and soon thereafter of meglumine antimoniate, no comparable drugs against CL have been discovered and developed.

Afghanistan has the highest incidence of CL caused by *L. tropica* or *L. major* in the world. Around 113 100 to 226 280 cases are estimated in Afghanistan each year [5, 6] and 4000 cases in the Leishmania and Malaria Centre in Mazar-e-Sharif alone [7, 8].

The economic impact of sodium stibogluconate (SSG) treatment [9–11] and drug related side effects of SSG treatment [12] lead to compliance failure, which in turn increase drug resistance [13]. In fact, the painful administration, especially for children, lead to patients drop out of around 59% to 37%, as was reported in clinical trials [14]. Compliance failures are supposedly much higher in non-experimental health care settings. Recent findings indicate genetic drug resistance to SSG in *L. infantum* parasites [13, 15].

Because of the disadvantages of a chemotherapeutic approach to CL cure, physical treatment procedures have also been studied in the literature [16]: thermotherapy, cryotherapy, surgery, electrotherapy, laser therapy, and photodynamic therapy. Although based on a parasitic aetiology, CL ulcers are open for more than 8 weeks and are therefore categorized as chronic [17]. Chronicity of CL wounds is due to a deficiency of the human immune system to cope with the parasites and to re-establish the natural wound healing process [18].

A phase IIa double-blind randomized controlled trial from 2002 to 2008 at the German Medical Service clinic in Kabul with *L. tropica* patients has shown that one application of bi-polar high-frequency electro-thermo-debridement (HF ETD) of the lesion under local anesthesia is an effective method leading to complete elimination of the parasite load in the scar after primary healing in 60% of the cases [19]. Moreover, the mean days for primary wound closure after start of treatment obtained in the phase IIa trial in Kabul was 42.6 (*SD* ± 2.87) days and shorter than the median time of 75 days for intra-dermal Sodium Stibogluconate (IL SSG) in a trial conducted by Reithinger et al. in Kabul [14].

On the basis of the above clinical results, a direct comparison between HF ETD with subsequent moist wound treatment (MWT) with DAC N-055 versus IL SSG within one single trial was considered necessary as a basis for further evidence-based decision-making. The Cochrane meta-analysis of González et al. [20] found no randomized controlled trials (RCTs) on the use of wound healing to treat OWCL. This led to the introduction of MWT with DAC N-055 alone as a third regimen in the Phase IIb RCT, without any previous physical debridement treatment [3].

The objective of the present investigation was to evaluate the efficiency of wound debridement and MWT regimens in direct comparison to mainstay IL SSG injections in CL patients in a mixed population of *L. tropica* and *L. major* parasite species in Afghanistan.

In a first step, a phase IIb RCT was designed and conducted to evaluate the efficacy of the investigated interventions compared to IL SSG. The clinical results have been published in Stahl et al. [3]. In summary, 87 patients were enrolled in the trial and were randomized into group I (*n* = 24), II (*n* = 32) and III (*n* = 31). The PP analysis of 69 (79%) patients revealed complete epithelialisation before day 75 in 15 of 23 (65%) patients of Group I, in 23 of 23 (100%) patients of Group II, and in 20 of 23 (87%) patients of Group III (*P* = 0.004, Fisher’s Exact Test). In the PP analysis, wound closure times were significantly different between all regimens in a pair-wise comparison (*P* = 0.000039, Log-Rank [Mantel-Cox] test). Re-ulcerations in patients of Group I, II or III respectively were not statistically significantly different (*P* = 0.312, Pearson Chi-Square Test). The clinical results are the basis for the efficacy model parameters of the present cost-effectiveness analysis as detailed in Additional file 3: Table S2.

In a second step, resources used were identified, quantified and valued on a patient level for all three regimens parallel to the evaluation of the clinical outcomes, compared in an incremental analysis. Sensitivity analyses were conducted to investigate robustness of results to parameter variations. A budget impact analysis was conducted in order to assess the impact in terms of cost of the alternative regimen from a societal perspective in the context of Afghanistan.

## Methods

### Ethical clearance

Ethical clearance of the health economic evaluation was obtained by the Ethical Committee of the Medical Faculty of the University of Heidelberg, Germany (S-318/2009, 18th November 2009) and the International Review Board at the Ministry of Public Health in Kabul, Afghanistan (23rd January 2010). The trial was registered online at Clinicaltrials.gov (ID: NCT00996463, 15th October 2009). All data used was anonymous. Ethical clearance was obtained from the IRB at the Ministry of Public Health that the informed consent could be obtained orally (low literacy rates) and documented in the electronic case report form. The German Federal Ministry of Education and Research sponsored the trial (Grant N° AFG 08/002).

### Target population and subgroup

The target population was defined by the patients’ inclusion and exclusion criteria detailed in the clinical and health economic trial protocol published by Stahl et al. [3]. All patients were used to parameterize the health economic analytical model. Subgroup analysis was not performed due to the small sample size of the underlying RCT.

### Setting and location

The health economic evaluation was based on cost and efficacy data on patient level collected during the RCT conducted at the Leishmaniasis and Malaria Centre (LMC) in Mazar-e-Sharif, Afghanistan, part of the Provincial Civil Balkh Hospital complex located in the city centre. *L. tropica* and *L. major* are the two *Leishmania* parasite species endemic in Mazar-e-Sharif, located in the northern Balkh Province of Afghanistan.

### Study perspective

The health economic study takes the societal perspective, including direct medical cost, direct non-medical and indirect cost. CL treatments are also routinely performed in villages, in environments with very basic health care facilities, if any. The identification of treatment resources in treatment Groups I to III was therefore limited to the most necessary items. This was done to avoid an over-estimation of treatment resources needed in the context of one of the poorest country in the world.

### Comparators

Based on the randomization, the CL lesion of the patients was treated either by IL SSG injection (Group I), by wound debridement with HF ETD with subsequent MWT with DAC N-055 (Group II) or by MWT alone with DAC N-055 (Group III).

### Visit protocol

Patients of all three regimens were scheduled to come to the LMC on a daily basis (with the exception of Fridays) during the first week of the treatment, followed by visits to the LMC twice a week until the end of week 4 and thereafter once a week until wound closure. Their scars were checked post closure once a month until 6 months had passed after the treatment was started.

### Treatment protocol

For patients in Group I, 0.6 ml (60 mg) SSG (Albert David Ltd., India) was administered 12 times by intra-dermal injections based on a protocol defined by Zeglin [21], differing from WHO IL SSG standard treatment protocol [17]. For patients in Group II, the intact skin surrounding the lesion was cleaned and disinfected. Following local anaesthesia, the wounds were debrided with HF-ETD. Subsequently, the wounds were dressed with jelly containing DAC N-055 (See Formula I in Stahl et al. [3]). Wound dressings were replaced daily. From the week 2 onwards patients were treated with the EuRho® DAC 2003 cream (Euro OTC Pharma GmbH, Bönen, Germany) until wound closure (See cream Formula II in Stahl et al. [3]). For patients in Group III, MWT was performed with DAC N-055 mixed into EuRho® DAC 2003 cream (See Formula II in Stahl et al. [3]). In the first week, dressing changes were performed during visits to the LMC in Groups II and III. Patients and their relatives were trained to change the wound dressings. Patients received 20 g EuRho® DAC 2003 cream containing DAC N-055 in a sterile syringe. During the visits at the LMC, the wound dressings and the healing progress were documented and recorded with digital pictures.

### Time horizon

CL caused by *L. major* or *L. tropica* is supposed to be a self-healing disease within the first year [1]. Resources used were documented only during treatment and follow-up visits within the Phase IIb RCT. It was assumed that no costs were associated with the epithelized lesion after final wound closure, since patients tend to be immune against re-infections [22].

### Discount rate

No discount rate neither on costs nor effects was applied due to the time horizon of 1 year.

### Choice of health outcomes

Although health related quality of life and cosmetic results of medical interventions are important in chronic CL lesions, days until wound closure are the most essential efficacy outcome for interventions in CL patients [23]. In the present health economic evaluation, health outcome was measured in wound free days (WFD), assuming that CL is a self-healing disease within 303 days. The duration of the non-treated open lesion was calculated based on literature [1] and a triangular distribution assumption. Ulcer free days were calculated as the difference in days between the self-healing time of CL lesions and the days for wound closure. In a previous study Stahl et al. [3] measured days for primary closure as the time in days starting with the first treatment administration until the day of primary closure documented with a digital picture. Days for wound closure were calculated as the sum of days for primary closure and in case of occurrence, the re-ulceration time in days.

### Measurement of effectiveness

The decision analytical model measures effectiveness as final wound closure time in days after eventual re-ulceration [2]. The follow-up period of up to 6 months also identified patients with re-ulcerations and no final closure, but without statistical significant difference between the regimens [3].

The Phase IIb RCT in Mazar [3] was designed as a mono-centric, controlled, randomized (1:1:1), open label and phase IIb health economic trial, collecting data on the clinical efficacy and resources used. For ethical reasons, the two-stage adaptive drop-loser sample size calculation was based solely on clinical endpoints. The results of the Phase IIb RCT [3] confirmed data from the Phase IIa RCT in Kabul [19] based on a sample size of more than 100 CL patients. There is enough evidence that the single efficacy and health economic study is a sufficient source of clinical effectiveness data, despite the small sample size of 23 patients in each arm. Final outcome of interest in the health economic evaluation is the incremental cost-effectiveness ratio of the two proposed regimen compared to the mainstay treatment with SSG.

### Estimating resources and costs

Direct medical resources used in each regimen were identified and quantified by the pre-defined clinical trial protocol and complemented by expert interviews by the local investigators. Valuation of the used items was conducted by averaging local market pharmacy prices. Direct medical costs were limited to the most necessary disposable items to administer the respective regimen. Equipment, furniture, room and personnel costs were not relevant in the incremental analysis. The identification of direct non-medical resources used was limited to time costs for the patient to reach the treatment centre and receive treatment. Indirect costs were assumed to be relevant. CL chronic wounds cause a decrease in productivity due to the open lesion. The quantification of the productivity decrease is an estimation based on an educated guess.

First we calculated the unit cost of each item by identifying, quantifying and then valuating each item used per injection or dressing. Thereby the average cost per dressing and the total dressing costs were calculated. Finally by adding fixed attributable costs per patient, we calculated the average cost per patient. In both regimens, Group II and Group III, wound dressings were administrated until primary wound closure. HF ETD was applied only once for each patient of Group II at the first day treatment start. In Group II and Group III, the dressings were changed during each visit and patients were taught to change their dressings by themselves at home. The average number of days between two dressings until wound closure was 3. Depending on the wound closure time, we could then calculate the total number of dressings and thereby based on average costs per dressing, we obtained the average total wound dressing costs for each patient. Direct medical cost of Group I followed a pre-defined treatment protocol as described by Stahl et al. [3].

Direct non-medical costs, e.g. travelling expenses, travelling time, waiting and treatment time, were recorded at each patient visit. Time costs were valued on the basis of 2013 per capita income in Afghanistan. Depending on the number of visits for each patient and the average non-medical costs for each visit, we could calculate the total average non-medical costs for each patient for each treatment option.

Indirect costs were calculated according to the human capital approach on the basis of the average 2013 per capita income in Afghanistan multiplied by the days necessary for primary closure of the lesion. After wound closure productivity loses were assumed to equal zero. All costs were calculated without value-added taxes, which are not existent in Afghanistan.

### Currency, price data, and conversion

Resource quantities of direct medical costs were calculated on the basis of the clinical trial protocol and collected alongside the trial between 2009 and 2011. Direct non-medical resources used were documented during the entire trial period between 2009 and 2011 for each patient. The productivity loss, that is indirect medical resources used, was estimated. Quantities were valued on the basis of Afghanis and if applicable German prices in 2013. Afghanis (Afs) and Euros (€) were converted to American Dollars (US$) with the average exchange rates of 2013 of the respective central banks. To update resources valued in monetary terms to a respective year subsequent to 2013 or make cost comparable to cost of other publications, present costs valued in US$ of for year 2013 need to be adjusted for inflation up to the year of interest, converted into local currency with the current exchange rate and converted into purchasing power parities of the respective year.

### Choice of model

Although a simple 3-stage Markov model describing the wound healing process of chronic wounds [24] would have been an attractive framework for the health economic evaluation of the study, the RCT did not include pre-defined recurrent health states. The decision problem involves a continuous risk over time of primary epithelisation and re-ulceration until final lesion closure. Timing of the lesion closure is an important characteristic of the chronic wound healing process as well. However, in the observation period of up to 180 days the RCT, re-ulceration only happened once. Therefore, instead of a Markov model, a decision analytical model was used to describe the health states identified during the clinical trial and formalized in a decision tree model [25].

### Model assumptions

One central assumption of the health economic model is that the disease is self-healing [1] and not lethal. In case of non-compliance or unsuccessful treatment, the self-healing time of the chronic CL lesion did not alter. Assumption over resource consumption in non-compliant patients was estimated as an educated guess fraction of average resources used for primary closure. Percentage productivity loss had to be estimated with an educated guess and assumed equal in all three regimen. Finally, the health economic model did not account for costs of side effects or complications such as infections due to the health care provision or the administrated treatments. In case of parasitic resistance to IL SSG or ulcers with no final closure after re-ulceration, the maximum number of injections and the SSG dosage was not increased. In contrast, in Group II and III DAC N-055 MWT dressings were administered until final closure of the CL ulcer. Patients were not re-assigned to another regimen in case of resistance or wound closure failure before self-closure of the CL lesion. Concerning the number of lesions per patient and the rate of productivity loss, no probabilistic distribution was defined due to lacking clinical evidence. The model needs to assume simultaneous wound closure, if simulated with more than one lesion. There are missing socio-economic investigations of indirect costs in chronic CL ulcers [26, 27].

### Study parameters

The baseline study parameters, the sensitivity ranges, and the distribution used in the decision tree are detailed in Additional file 3. Additional file 3: Table S1 contains the parameters used to calibrate the decision nodes. Additional file 3: Table S2 detail the efficacy parameters. Additional file 3: Tables S3-S5 detail the direct medical, the direct non-medical and the indirect cost of the three regimens.

The baseline nodes probabilities for all three regimens were based on the patient data from Stahl et al. [3]. Sensitivity ranges were defined as ±10% deviations from baseline values. In the probabilistic sensitivity analysis, the parameters of the beta distributions are defined according to the number of patients in the clinical trial in the respective pre-defined and photo-documented health states.

The efficacy parameter of the regimens result from the RCT [3]. Sensitivity ranges were defined in most cases with obtained minimum and maximum values. The small sample size restricted the use of confidence intervals to the sensitivity ranges of mean days for primary closure parameters and constituted the rationale for using the triangular distribution within probabilistic analyses. The triangular distribution parameters were based on the respective minimum and maximum sensitivity ranges of each parameter. The modus of the triangular distribution was chosen such that the baseline parameter value resulted as mean from the parameterized triangular distribution.

Within the direct medical cost, the baseline unit price of 8.5 cent per gram for the preparation of the magistral DAC N-055 basic crème preparation according to the formula detailed in Stahl et al. [3] was solely based on the tax-exempted wholesale prices of the ingredients. The DAC N-055 jelly price equals that of the basic crème (see Stahl et al. [3] for DAC N-055 basic crème formula). Sensitivity ranges were defined as plausible minimum and maximum unit price values for 2013. The final price depends on the additional cost for the DAC N-055 basic crème preparation in pharmacies or companies with good manufacturing production (GMP) license and all additional cost associated with commercialization. Probabilistic sensitivity analyses were conducted on the basis of a gamma distribution around the baseline DAC N-055 basic crème price. Data to quantify direct non-medical cost of CL treatments were collected at each patient visit during the RCT. Parameters represent average values. Baseline indirect cost, e.g. productivity losses due to the disease, was assumed to average 1% reduction of labor productivity until wound closure. Since after CL infections women might not be allowed to cook, to have children or to marry in Afghanistan [27], the indirect cost of CL ulcers and especially the indirect cost of persisting CL scars were underestimated in the present health economic evaluation. For women in Afghanistan, marriage is still the main income security. Nonetheless, the sensitivity range was conservatively defined to vary from 1 to 20% to account for comparability with variations of other variable values within the tornado diagram analyses.

### Analytic methods

The decision tree was calibrated based on probabilities of health states within the RCT, which are the compliance, primary closure, re-ulceration and final closure after re-ulceration. Four different final health states were modeled and defined according to their costs and efficacy outcome. The decision tree is represented in Additional file 4. The health economic model was simulated with TreeAge Pro 2014 (TreeAge Software, Inc., One Bank Street, Williamstown, MA 01267, USA) with the parameter values reported in Additional file 2. Study baseline parameters were calculated on the basis of the RCT primary patient level data collected [3]. Sensitivity ranges were calculated on the basis of minimum, maximum values, confidence intervals or ±10% deviations. Minimum, maximum values were taken for parameters with small samples. Resource prices were assumed to vary up to ±10%. Confidence intervals were calculated for efficacy data. Distributions were defined for the different parameters according to the data properties. Costs were typically left skewed distributed and followed a gamma distribution. Data defined by their minimum, maximum and modus properties followed a triangular distribution. Decision paths probabilities calculated on the basis of the fraction of patients within that health state were calibrated according to the beta distribution. All other parameters were defined as normally distributed [28]. According to WHO-CHOICE criteria [10] a health intervention is very cost-effective if the incremental cost-effectiveness ratio (ICER) is below the respective per capita gross domestic product (GDP), cost-effective if between one to 3 times the per capita GDP of a country, and not cost-effective if above 3 times the per capita GDP. In Afghanistan, the per capita income per day was around US$ 1.92 in 2013. The incremental cost per incremental WFD of the health interventions should be below that US$ 1.92 threshold to be very cost-effective. Therefore, the net monetary benefit (NMB) corresponds to the threshold (WTP) of US$ 1.92 multiplied by the medical outcome measured in natural units (WFD) minus the resources used valued in monetary units (Cost). In this case the threshold is defined as willingness-to-pay (WTP) per natural outcome and in the present cases monetarily values a WFD with US$1.92. The analysis was conducted on the assumption that the three regimens were mutually exclusive. A baseline incremental cost-effectiveness analysis, a budget impact analysis, uni- and multivariate sensitivity analyses, and a probabilistic Monte-Carlo simulation with 10 000 iterations were conducted with the TreeAge software.

## Results

### Baseline evaluation

**Fig. 1:**
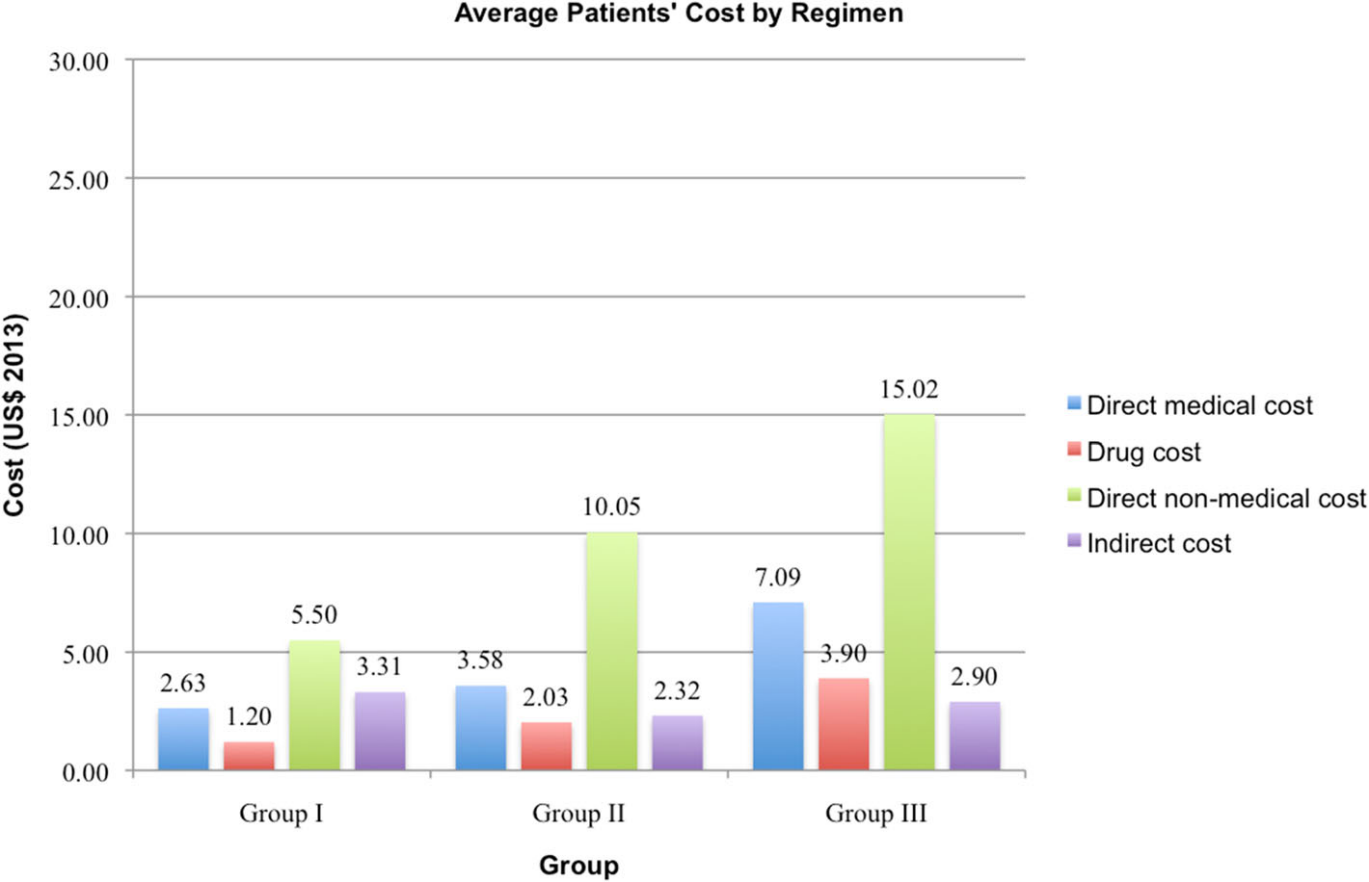
Average Cost per Patient by Regimen. Comparison of direct medical, drug, direct non-medical, and indirect cost per patient for each regimen

The baseline cost-effectiveness analysis in Fig. 1 corresponds to average treatment costs and WFD for patients with the baseline patients’ clinical characteristics detailed above with one CL lesion. The average costs and WFD values given in Table 1 are weighted by their probability of occurrence in each pre-defined health state. The mean costs of US$ 15.91 (*SE* = 7.58) in Group II and US$ 24.97 (*SE* = 0.58) in Group III are higher than the costs of US$ 11.43 (*SE* = 0.016) in Group I. Regular dressing changes to promote the lesion’s closure and avoid super-infections increase the average cost of treatment in Group II and Group III by US$ 4.48 and US$ 13.54 respectively compared to IL SSG injections with no adjuvant wound care until final closure. Baseline effectiveness in wound free days are 177 (*SE* = 0.36) in Group II, 147 (*SE* = 0.33) in Group III, and 129 (*SE* = 0.27) in Group I. Group II versus Group I has the highest incremental effectiveness of 48 WFD compared to 17 incremental WFD in Group III versus Group I. The effectiveness findings are based on the simulation of the calibrated decision tree. The simulation findings reflect the results from the underlying clinical RCT [3] and thereby validate clinically the health economic model used. When compared to Group I, the ICER of Group II of US$/WFD 0.09 and US$/WFD 0.77 of Group III are below an Afghan per capita income threshold of US$ 1.92 per day in 2013. In a mutually exclusive comparative cost-effectiveness analysis against Group I, Group II is more efficient than Group III. Given the resources used included in the analysis, all three regimen have a positive net monetary benefit (NMB) as stated in Table 1 assuming a WTP of US$/WFD 1.92 in 2013: US$ 236 (*SE* = 0.51) in Group I, US$ 324 (*SE* = 7.6) in Group II, and US$ 257 (*SE* = 0.80) in Group III. Given the baseline parameter, remarkably the total cost share of direct medical cost is half that of direct non-medical cost per patient. Half of direct medical costs are drug costs, which roughly average to US$ 4 in the WHO-EMRO IL SSG protocol [17] compared to US$ 1.20 in the IL SSG protocol defined by Zeglin [21] and tested in Stahl et al. [3].

**Table 1:** Cost and effectiveness with baseline parameters

	Health states					Av. Total
	Non-compliant	Primary closure	No primary closure	Final closure	No final closure	
Probability of health states						
WHO EMRO protocol^a^	0.39	0.48	0.03	0.08	0.03	1.00
Group I	0.39	0.48	0.03	0.08	0.03	1.00
Group II	0.28	0.62	0.01	0.06	0.03	1.00
Group III	0.26	0.51	0.01	0.06	0.16	1.00
Direct medical cost						
Group I (US$)	0.87	3.48	3.48	4.93	4.93	2.63
Group II (US$)	0.81	3.26	28.31	6.26	23.40	3.58
Group III (US$)	1.05	4.21	28.14	8.02	24.53	7.09
Injections/dressings						
WHO EMRO^a^ (*N* =)	2	8	8	13	13	6
Group I (*N* =)	3	12	12	17	17	9
Group II (*N* =)	3	11	101	21	72	12
Group III (*N* =)	4	15	101	28	73	23
Drug cost						
WHO EMRO^a^ (US$)	1.32	5.28	5.28	8.58	8.58	4.11
Group I (US$)	0.40	1.58	1.58	2.24	2.24	1.20
Group II (US$)	0.47	1.87	17.17	3.57	12.24	2.03
Group III (US$)	0.64	2.55	17.17	4.76	12.41	3.90
Direct non-medical cost						
Group I (US$)	1.36	7.70	7.70	9.97	9.97	5.50
Group II (US$)	1.88	10.26	69.22	17.57	49.59	10.05
Group III (US$)	2.23	11.29	60.12	19.35	43.47	15.02
Indirect cost						
Group I (US$)	5.82	1.32	5.82	1.61	5.29	3.31
Group II (US$)	5.82	0.63	5.82	1.64	4.80	2.32
Group III (US$)	5.82	0.86	5.82	1.61	5.07	2.90
Total average cost						
Group I (US$)	8.05	12.50	17.00	16.51	20.19	11.43
Group II (US$)	8.51	14.15	103.35	25.47	77.79	15.91
Group III (US$)	9.10	16.36	94.08	28.98	73.07	24.97
Wound free days						
Group I (WFD)	0	234	0	207	0	129
Group II (WFD)	0	270	0	167	0	177
Group III (WFD)	0	258	0	233	0	147
ICER						
Group I (US$/WFD)	N.A.	0.00	N.A.	0.00	N.A.	0.00
Group II (US$/WFD)	N.A.	0.05	N.A.	−0.22	N.A.	0.09
Group III (US$/WFD)	N.A.	0.16	N.A.	0.48	N.A.	0.77
NMB (WTP = 1.92)						
Group I (US$)	−8	437	−17	381	−20	236
Group II (US$)	−9	504	− 103	295	− 78	324
Group III (US$)	−9	479	−94	418	−73	257

All costs in 2013 US$

^a^ WHO EMRO protocol is defined as 1 to 5 ml IL SSG, twice weekly for three to 4 weeks [4]. The probability of health states of Group I was also assumed for the IL SSG WHO EMRO protocol, as a best guess. We assumed a mean IL SSG dosage of 3 ml per injection and 5 additional injections for final closure based on the IL SSG re-ulceration time found in Stahl et al. [3]

### Heterogeneity

Heterogeneity was not simulated in the health economic model due to the non-significant difference in the underlying patient population of the Phase IIb RCT [3]. However, the number and size of lesions per patient, the parasite specie, the patients’ age and gender should be investigated in future treatment evaluations to characterize heterogeneity. This was not possible regarding the sample size of the underlying clinical study.

### Cost sensitivity

Changes in parameter values show different parameter sensitivities of costs (see Additional file 4: Figures S1-S6). Moreover the extent of changes in average total cost depends also on the ranges of variation of the model parameters (see Additional file 3). Some parameters only change in steps of 100% and above to impact average cost, like for example the number of lesions or the total number of injections. Obviously, the sensitivity of average costs in Group I to parameter value variations is less important than in Group II and III. In Group I, the most sensitive cost parameters are the number of lesion, the SSG unit price, and the duration of the natural wound healing, in Group II and III the DAC N-055 unit price, the number of lesions, and the average days between two dressings, and additionally in Group II the probability of primary closure.

### Drug price

**Fig. 2:**
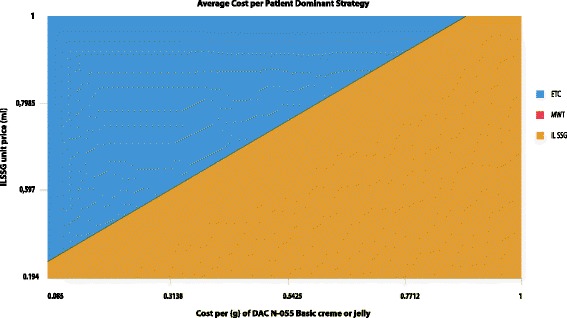
Three-way Sensitivity Analysis of Drug Pricing. Variations of unit drug price of DAC N-055 basic crème or jelly and SSG with an average 2.75 ml dosage of SSG per lesion according to WHO-EMRO case management guidelines [19]

Figure 2 shows the comparative patient costs of the three regimens as a function of DAC N-055 and SSG unit drug price with an average IL SSG dosage of 2.75 ml per injection per lesion for an average number of 9 injections according to the WHO EMRO case management guideline [4]. In our model, average patients’ cost in Group III (red color) will always be higher than average patients’ cost in Group II or III, independently of price variations in either drug. However, patients’ costs in Group II (blue surface) are lower than in Group I (brown surface) depending on the price relation between SSG and DAC N-055.

### Sensitivity of direct non-medical cost

Independently of the regimen, the frequency of visits, including follow-up visits, increases average cost by US$ 3 to 10. The share of direct non-medical costs can be 24 to 67% of total average costs. Table 3 shows that the cost variations are small in Group I, but a reduction of the number of visits in Group II or III from 12 to 6, impacts the INMB of the wound care regimens compared to IL SSG by US$ 3 to 4 per patient, which corresponds approximately to average SSG drug cost per patient according to the WHO EMRO protocol [4].

### Sensitivity of indirect cost

Loss of productivity, defined as indirect costs, is calculated in the simple decision analytical model with the time the CL ulcers is either open or re-ulcerated until final closure. Table 3 shows the effect of differences in the percentage of productivity loss on the choice of the dominant cost-effective regimen. A productivity decrease from 1 to 10% has approximately the same absolute impact on average patient cost of Group II and III as a unit price increase of DAC N-055 basic crème by around 1000% (See Table 3 and Additional file 4: Figures S2 and S3). In case productivity loss solely in Group I increased from 1 to 10%, both alternatives of Group II and III would dominate the treatment strategy of Group I. Accurate quantification on the impact of chronic wounds on productivity and income, especially for women in case of CL, even after wound closure, are sparse as our yet unpublished systematic literature review has shown [26].

### Budget impact analysis

The budget impact analysis of Table 2 considers the additional resources needed to provide the proposed treatments, without quantifying or valuing the effectiveness of the regimens. The baseline budget impact analysis was calculated for Afghanistan on the basis of the estimated CL incidence data published by Alvar et al. [5]. The budget impact analysis has the limitation to consider CL patients with only one lesion, due to the evidence collected within the underlying RCT [3]. Based on average incidence, comparison of Group II and III to Group I result in additional societal cost of around US$ 0.765 and 2.301 million respectively. In case of non-treatment, direct resources are not used, but economic relevant indirect costs still have to be considered. The budget impact of the no-treatment alternative is US$ 879 875, which is higher than the drug cost of the WHO-EMRO guideline IL SSG regimen in Group I of US$ 697 395, and higher than the drug cost of US$ 343 650 in Group II and US$ 660 963 in Group III. When indirect costs are considered, the “no treatment” alternative is no sustainable option. This result can be confirmed in a NMB calculation of the treatments, when outcome of respective CL treatments are valued in WTP, as detailed in Table 1.

**Table 2:** Budget impact analyses with baseline parameters

	Number of patients (*N*)^a^			
	WHO EMRO protocol	Group I	Group II	Group III
Health states				
Non-Compliant	65 500	65 500	47 683	43 780
Primary closure	81 621	81 621	104 868	86 712
No primary closure	4582	4582	1358	1358
Final closure	13 575	13 575	10 521	10 860
No final closure	4582	4582	5260	27 150
	Budget impact (US$ 2013)^a^			
	No treatment	Group I	Group II	Group III
Health states				
Non-Compliant	–	527 278	405 781	398 398
Primary closure	–	1 020 261	1 483 888	1 418 602
No primary closure	–	77 888	140 300	127 715
Final closure	–	224 127	268 069	314 727
No final closure	–	92 503	409 206	1 983 880
Total				
Av. incidence (169 860 cases)	879 875	1 942 056	2 707 244	4 243 322
Min. incidence (113 100 cases)	585 858	1 294 399	1 804 404	2 828 215
Max. incidence (226 280 cases)	1 172 130	2 589 714	3 610 085	5 658 430
NMLCP rep. Incidence (22 620 cases)	117 172	258 880	360 881	565 643
	Drug cost (US$ 2013)^a^			
	WHO EMRO protocol	Group I	Group II	Group III
Health states				
Non-Compliant	86 460	25 938	22 292	27 910
Primary closure	430 958	129 287	196 104	221 115
No primary closure	24 191	7257	23 309	23 309
Final closure	116 475	30 463	37 559	51 694
No final closure	39 310	10 281	64 387	336 936
Total				
Av. incidence (169 860 cases)	697 395	203 227	343 650	660 963
Min. incidence (113 100 cases)	464 821	135 453	229 046	440 538
Max. incidence (226 280 cases)	929 970	271 001	458 254	881 388
NMLCP rep. Incidence (22 620 cases)	92 964	27 091	45 809	88 107

^a^ The budget impact calculations for the average estimated incidence differentiated by health states is calculated with the total average cost per patient with one lesion and the drug cost for each regimen. Drug costs represent solely the costs of SSG and DAC N-055 basic crème according to the pre-defined dosage and posology. The number of patients are based on estimated average incidence published by Alvar et al. [5] and the probability of final health states used in the present decision tree model. In case “no treatment” is administered, based on Afghan 2013 per capita income of US$ 700 and an average lesion duration of 303 days, according to our simple model and the educated guess, indirect cost due to one CL lesion amount to US$ 5.81. If additionally, productivity loss due to CL exceeds 1% of daily income, the societal budget impact of “no treatment” is considerable

Depending on the number, size, site and severity of CL ulcers, productivity losses can be assumed to be higher than 1%, especially for children and women, in case when life-long stigmatization costs of CL disfiguring scars are taken into account. To calculate the disability adjusted life years (DALYs), the disability weight (DW) for CL used by WHO equals 0.023 or 2.3% per year. The indirect cost based on the productivity loss of 1% is therefore an underestimation compared to the CL disability weight used in DALY calculations.

### Outcome sensitivity

Additional file 4: Figures S4-S6 represent the absolute variations in effectiveness in average WFD based on predefined parameter variations. The most outcome-sensitive parameters are the duration of the disease, the probability of treatment compliance, the probability of primary closure. However, variations in effectiveness of Group III additionally depend on the probability of re-ulceration.

### Three way-sensitivity analysis

Parasitic resistance and patient compliance due to potential pain and toxic side effects of IL SSG regimen are the most important parameters to investigate comparative effectiveness of alternative regimens to treat CL as shown in Additional file 4: Figure S7 and S8. The probability of primary closure in Group I was interpreted as proxy for parasitic resistance. Moreover, the SSG regimen has side effects that, although very rare, can potentially be fatal (anaphylactic shock and cardio-toxicity), especially in children and elderly persons, besides the repeatedly painful administration. The probability of compliance in SSG regimen can be interpreted as proxy for the adverse effects of therapy. The 3-way sensitivity analysis investigates the dominant regimen with respect to WFD as a function of the probability for primary closure in Group II versus Group I versus the baseline parameters of Group III in dependence of the probability of compliance in Group I (12.5% versus 25% non-compliant patients (Additional file 4: Figures S7 and S8, respectively)).

At a compliance rate of 75% in Group I and a 100% primary closure probability in Group I, wound debridement with subsequent wound care in Group II is the most effective treatment given baseline parameters (blue surface). Below a probability of primary closure in Group II of 82 and 89% in Group I, simple MWT shows the highest effectiveness, given baseline parameters. The shift in the probability of compliance in Group I from 75 to 87.5% increases the probability of dominance in effectiveness of Group I (compare brown surface in Additional file 4: Figures S8 and S7).

### Sensitivity of incremental cost-effectiveness ratios

The ICER_II,I_ comparing Group II versus Group I varies up to US$ 0.47 due to uni-variate unit drug price increase of Group II from US$ 0.085 to 1.0. In Group II, the probability of primary closure (90 to 100%), the number of lesions (1 to 4), and the average days between two dressings (2 to 4 days) impact the most the ICER_II,I_ variation: US$ 0.23, US$ 0.22, and US$ 0.18, respectively.

In contrast, the ICER_II,I_ spreads only by US$ 0.05, if the days for primary closure in Group II change within the confidence intervals of 29 to 37 days obtained in the underlying RCT [3]. Variations in the probability of compliance in Group II as defined in the Additional file 3 within the ±10% sensitivity ranges, affect the ICER by up to US$ 0.04. All ICER_II,I_ are all below the WTP threshold of US$ 1.92.

The ICER_III,I_ is sensitive to Group I parameter variations in the probability of treatment compliance (spread US$ 2.412), to the absolute days for primary closure (spread US$ 1.252), and the probability of primary closure (spread US$ 0.734).

The ICER_III,I_ is sensitive to Group III probability of treatment compliance (spread US$ 3.739), Group III probability of primary closure (spread US$ 3.476), to the DAC N-055 drug unit price (spread US$ 2.65), and the number of lesions in Group III (spread US$ 1.207).

### Probabilistic sensitivity analysis

#### Cost-effectiveness plane and acceptability curve

**Fig. 3:**
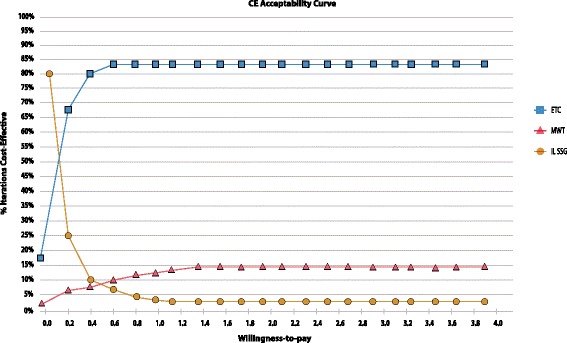
Cost-Effectiveness Acceptability Curve. Cost-effectiveness acceptability curve resulting from a probabilistic sensitivity analysis with 10 000 iterations in a Monte Carlo simulation

Stochastic uncertainty was explored within a probabilistic sensitivity analysis on the basis of all the defined distributions and calculated distribution parameter values. The cost-effectiveness plane of Group II and Group III versus Group I and Group III versus Group II are represented in Additional file 4: Figures S9-S11. A Monte-Carlo simulation with 10 000 iterations allowed calculating the probability for each intervention to be cost-effective depending on a willingness-to-pay threshold. The results are represented graphically in the cost-effectiveness acceptability curve (CEAC) in Fig. 3. Group II is the most cost-effective strategy in 80% of the cases starting with a WTP of about 80 cents. Group III is only the most cost-effective strategy in 10% of the cases at a WTP of 80 cents per WFD. If patients or society does not value wound free days with a willingness-to-pay, then the most cost-effective treatment is IL SSG in over 90% of the cases, since the average cost per patient for Group I is the lowest. The median costs and outcomes of the Monte-Carlo simulation confirm baseline results.

## Discussion

### Study findings

Table 1 shows that ETD with MWT is the most cost-effective treatment compared to standard IL SSG treatment. MWT is the second best treatment alternative compared to standard IL SSG treatment. The budget impact analysis of Table 2 shows that societal costs of non-treatment are higher than the drug cost of either treatment. Economic evaluations of treatment alternatives focusing only on drug or direct medical cost, lead to misallocation of resources.

On the side of resources used, direct costs and indirect cost amount only to around one quarter of total cost. Direct non-medical costs amount to half of baseline treatment costs. Table 3 shows how strong variation in the number of visits and in productivity loss impact average treatment cost. On the side of effectiveness, probability of compliance, primary closure, and re-ulceration are the most important effectiveness-sensitive parameters to monitor during the implementation of the proposed regimen. The baseline results are robust to different sensitivity analysis.

**Table 3:** Sensitivity analysis on the impact of the number of visits at the treatment centre on average cost per patient and on the impact of productivity loss on average cost per patient

	Av. days open CL ulcer	Av. cost per patient	Av. direct non-medical cost per patient		NMB (WTP = 1.92)	INMB (WTP = 1.92)			
			% of av. cost	Abs. value		Group II: 6 visits	Group II: 12 visits	Group III: 6 visits	Group III: 12 visits
Group I									
6 visits at treatment centre	174	10.13	27	2.71	238	90	86	29	25
9 visits at treatment centre	174	11.43	36	4.07	236	92	88	31	27
12 visits at treatment centre	174	12.50	43	5.42	235	93	89	32	28
Group II^a^									
6 visits at treatment centre	126	11.82	35	4.09	328	0	−4	−61	−65
9 visits at treatment centre	126	13.87	44	6.14	326	2	−2	−59	−63
12 visits at treatment centre	126	15.91	51	8.18	324	4	0	−57	−61
23 visits at treatment centre	126	23.42	67	15.69	316	12	8	− 49	−53
Group III^a^									
6 visits at treatment centre	156	14.88	24	3.56	267	61	57	0	−4
9 visits at treatment centre	156	16.66	32	5.34	265	63	59	2	−2
12 visits at treatment centre	156	18.44	39	7.12	263	65	61	4	0
23 visits at treatment centre	156	24.97	55	13.65	257	71	67	10	6
	Av. days open CL ulcer	Av. cost per patient	Av. indirect cost per patient		NMB (WTP = 1.92)	INMB (WTP = 1.92)			
			% of av. cost	Abs. value		Group I: 1% prod. Loss	Group I: 10% prod. Loss	Group I: 20% prod. Loss	
Group I									
1% productivity loss	174	11.43	28.96	3.31	236	–	30	63	
10% productivity loss	174	41.19	80.38	33.11	207	−30	–	−33	
20% productivity loss	174	74.26	89.17	66.22	174	−63	−33	–	
Group II									
1% productivity loss	126	15.91	14.59	2.32	324	88	118	151	
10% productivity loss	126	36.60	62.79	22.98	304	67	97	130	
20% productivity loss	126	59.58	77.16	45.97	281	44	74	107	
Group III									
1% productivity loss	156	24.97	11.62	2.90	257	20	50	83	
10% productivity loss	156	51.10	57.73	29.50	231	−6	24	57	
20% productivity loss	156	80.12	72.50	58.09	202	−35	−5	28	

^a^ No changes have been made to the treatment protocol in each regimen, only the number of visits to the centre have been varied. Dressing changes can be done by the patient himself, visits at the centre can be reduced to a minimum check of the wound healing process. In Group I, 6 visits correspond to the WHO protocol, 6 visits to the average of visits including non-compliant patients, and 12 visits to the foreseen RCT protocol for IL SSG injections

The present health economic evaluation did not account for loss in expected “lifetime-income” of potential serious adverse event cases. The patients’ health risks of any kind of treatment should not be increased, especially when the disease is self-healing like CL. The indirect costs resulting from serious adverse events would probably additionally advocate against the on-going use of SSG in specific patient populations.

### Limitations

Productivity losses have to be evaluated more accurately, because the efficient allocation of resources within society depends on their quantification. Cost-of-illness (COI), quantifying the opportunity cost of CL being endemic in a country or region, were not accounted for in the evaluation of the three regimens. COI quantify not merely the cost of disease management, but the resources used compared to a situation where there is no disease. Therefore, COI include for example the opportunity cost of vector and disease surveillance, preparedness and control activities, effects on tourism, information, communication and education, besides the cost of social stigmatization, especially for women in Afghanistan. Treatment regimens can impact COI differently.

Moreover, the health economic model was based on a single Phase IIb RCT [3] with a relatively small sample size and clinical endpoints and patient characteristics involving one single CL lesion. Therefore, the model cannot account for primary data heterogeneity or subgroup analysis.

The follow-up period of future trials should be extended to 365 days to monitor re-ulceration rates and account for the self-healing assumption of CL within 1 year. Theoretical heterogeneity calculations with respect to the number of lesions per patient should also guide future research. Such evaluations should not be based necessarily on the assumption that the patients’ lesions close and eventually re-ulcerate simultaneously.

The authors did not find any RCT investigating the effectiveness of WHO IL SSG in dependence of dosage and posology. EMRO-WHO IL SSG dosage and posology recommendations [4] increase the average drug costs per CL patient. Moreover, the societal budget impact analysis did not include a probabilistic analysis. The authors assumptions is that shorter primary wound closure improves cosmetic outcome, especially of CL ulcers on the face. Therefore the treatment decision impact the long term costs of stigmatization, social exclusion of affected women and children [6]. However, the present model did not include indirect cost due to disfiguring scarring and the cosmetic outcome of treatment. In the majority of cases, CL treatment in Afghanistan is financed out-of-pocket. Therefore, indirect costs are decisive for the CL patient and relatives. The authors’ assumption of 1% productivity reduction on average is conservative considering the CL disability weight of 0.023 used in disability adjusted life years (DALYs) calculations.

### Generalizability

The health economic results obtained are applicable to the context of Afghanistan. The decision tree model can be calibrated with parameter values corresponding to the prices of other country settings to investigate the cost-effectiveness of the proposed interventions, assuming the reproducibility of the clinical results in other health care settings given the power and significance level of the sample size calculation in Stahl et al. [3].

The decision tree model was used based on the health states documented during the RCT. Future CL trials could document the clinical healing process of chronic wounds more dynamically by differentiating health states such as open lesion, debrided wound with or without tissue granulation with or without infection and bio-film, lesion closure, re-ulcerations, final closure. The clinically based health economic models in CL chronic wounds would need to be adapted accordingly.

The treatment approach of Group II is based on findings in the healing process of chronic wounds indicating that bio-films are a major source for bacterial proliferation and super-infections leading to complications in wound healing and days for complete epithelization [24]. The discussion of the present findings should be conducted through transparent scientific health economic evidence without conflict of interest.

### Current knowledge

The present economic evaluation of CL wound care regimen is the first published cost-effectiveness analysis conducted alongside a clinical trial in CL patients in Afghanistan. In a systematic literature review on the health economics of chronic wounds to be published by Leporowski et al. (Master thesis not yet published [26]), cost and effectiveness data was extracted from 66 publications from a total of 732 publications screened and published since 1966. The systematic review focused on health technologies for the treatment of chronic wounds with the highest annual incidence in western countries: venous and arterial ulcers, diabetic foot ulcers, and pressure ulcers. Leporowski et al. found ten cost-of-illness, five cost-minimization, 37 cost-effectiveness and 14 cost-utility-analysis. Although all costs were converted with purchasing power parity to IUS$ 2014 difficulties prevail to compare interventions with respect to their costs and effectiveness due to very different methodological approaches. The results of the systematic literature review [26] indicate that the resources and the outcome parameters considered in the present health economic investigation correspond to best scientific practices and the results are generate on the best available evidence.

## Conclusions

Jebran [19] showed the rationale of adjuvant application of MWT with DAC N-055 in wound care of chronic CL ulcers compared to simple adjuvant MWT with physiological saline, especially in CL lesions with *L. tropica* rest load after primary wound closure. The present health economic evaluation shows that adjuvant MWT in crusted chronic CL lesions covered by bio-films and infiltrated with *L. tropica* or *L. major* parasites is an efficient treatment option in the context of Afghanistan. However, average costs and effects in Group II have unexplained higher standard errors compared to Group I and Group III. The baseline results of adjuvant MWT and prior electro-thermo-debridement are robust against probabilistic sensitivity analyses. The comparative efficiency of Group III depends on the probability of treatment compliance, the probability of primary closure, and can be a sustainable efficient alternative to SSG or complement the IL SSG wound treatment depending on the clinical case as for instance children, women, or elderly. Decision makers should be aware, when allocating resources, that from a societal health economic perspective based on the budget impact analysis, the indirect cost and the NMB baseline calculations, aseptic MWT should always be offered to CL patients. Wasted resources are less and medical outcome are better compared to no-treatment of CL patients.

## Additional files

Additional file 1: Multilingual abstract in the five official working languages of the United Nations. (PDF 478 kb)

Additional file 2: Decision analytical model. Three treatment alternatives in patients with open chronic cutaneous Leishmaniasis wounds. (PDF 281 kb)

Additional file 3: Calibration of the decision tree with model assumptions and decision nodes probabilities, Efficacy parameters, Direct medical costs, Direct non-medical costs, Indirect costs. Baseline values, sensitivity ranges and distributions. (PDF 287 kb)

Additional file 4: Tornado diagrams, 3-way sensitivity analyses and Cost-Effectiveness Planes. In the 3-way Sensitivity Analysis of Effectiveness represent variation of primary closure probabilities in Group I versus II with a 12.5% (Figure S7) and 25% (Figure S8) non-compliant patients rate in Group I. (PDF 1082 kb)

## Abbreviations

BMBF: German Federal Ministry for Education and Research
CE: Cost-effectiveness
CL: Cutaneous leishmaniasis
DAAD: German Academic Exchange Service
DAC N-055: Pharmaceutical sodium chlorite listed in the German Drug Codex
DAC: German Drug Codex
DALY: Disability adjusted life years
EMRO: Eastern Mediterranean Regional Office
ETD: Electro-thermo-debridement
EV: Expected value
GDP: Gross domestic product
GMP: Good manufacturing production
HF: High-frequency
ICER: Incremental cost-effectiveness ratio
IL SSG: Intra-dermal Sodium Stibogluconate
INMB: Incremental net monetary benefit
ITT: Intention-to-treat
IUS$: International United States Dollars
*L. major*: *Leishmania major*
*L. tropica*: *Leishmania tropica*
LMC: Leishmaniasis and Malaria Centre
MoPH: Ministry of Public Health
MWT: Moist wound treatment
NMB: Net monetary benefit
NMLCP: National Malaria and Leishmaniasis Control Program
NTD: Neglected tropical diseases
PP: Per protocol
PPP: Purchasing power parity
RCT: Randomized controlled trial
*SE*: Standard error
SSG: Sodium Stibogluconate
US$: United States Dollars
WFD: Wound free days
WHO: World Health Organization
WTP: Willingness to pay
