# Supplementary material for: Health economic evaluation of moist wound care in chronic cutaneous leishmaniasis ulcers in Afghanistan
Source: Infect Dis Poverty. 2018 Feb 14;7:12. doi: 10.1186/s40249-018-0389-4 (PMC5812215; doi:10.1186/s40249-018-0389-4)
Supplement: Supplementary file 1 — Multilingual abstract in the five official working languages of the United Nations. (PDF 478 kb) [file 40249_2018_389_MOESM1_ESM.pdf]

Translation of the abstract into the five official working languages of the United Nations

التقييم الاقتصادي الصحي لرعاية الجروح الرطب في قرح داء الليشمانيات الجلدي المزمنة في أفغانستان

ستهل، هانز-كريستيان، أحمدي، فريد الله؛ نهضة، سامي محمد؛ دونج، هنجچين؛ شتال، كورت-ويلهلم وسويربورن، رينر

ملخص

خلفية: تهدف التقييمات الاقتصادية الصحية الحالية في أفغانستان إلى دعم صانعي القرارات في مجال الصحة العامة ومديري الرعاية الصحية لتخصيص الموارد بكفاءة للعلاج المناسب لداء الليشمانيات الجلدي الناتجة عن الليشمانيا المدارية أو الليشمانيا الكبيرة.

الطريقة: تم استخدام شجرة القرارات لتحليل تكلفة وفعالية نظامين للعناية بالجروح مقابل حقن الأنتيمون في الأوعية الدموية في المرضى من كل في أفغانستان. وقد جمعت التكاليف من منظور اجتماعي. تم قياس الفعالية في أيام خالية من الجروح. وقد حسبت النسبة الإضافية للفعالية من حيث التكلفة والمنفعة النقدية الصافية الإضافية. وكان النموذج معلما بمعلمات خط الأساس ونطاقات الحساسية وتوزيعات المعلمات. وأخيرا، تم محاكاة النموذج وتم تقييم النتائج بتحليل احتمالية وحساسية الاحتمال. وكانت النتائج النهائية هي كفاءة النظم وتحليل أثر الميزانية وفقا لأوضاع أفغانستان.

النتائج

بلغ متوسط التكاليف لكل مريض 11 دولارا أمريكيا (*SE*= 0.016) (المجموعة الأولى: ستيبوجلوكونات الصوديوم داخل الآفة)، و16 دولارا أمريكيا (*SE*= 7.58) (المجموعة الثانية: الكي الكهربائي الحراري+علاج الجروح الرطب) و25 دولارا أمريكيا (*SE*= 0.48) (المجموعة الثالثة: علاج الجروح الرطب) في المرضى الذين يعانون من قرحة واحدة ناتجة من داء اللايشمانيا الجلدي المزمن. ومن شأن أثر النظامين الثاني والثالث في الميزانية أن يوفر مبلغا قدره 0.353 مليون دولار أمريكي و0.036 مليون دولار أمريكي لتكلفة الأدوية على التوالي، مقارنة ببروتوكول إدارة الحالات في المنظمة. ويبلغ متوسط الفعالية في الأيام الخالية من الجروح 177 (*SE*= 0.36) في المجموعة الثانية، 147 (*SE*= 0.33) في المجموعة الثالثة، و129 (*SE*= 0.27) في المجموعة الأولى. وكان معدل فاعلية التكلفة التزايدية للمجموعة الثانية مقابل المجموعة الأولى 0.09 دولار أمريكي والمجموعة الثالثة مقابل المجموعة الأولى 0.77 دولار أمريكي، وهو فعال جدا من حيث التكلفة مع عتبة استعداد للدفع تبلغ 2 دولار أمريكي لكل يوم خالي من الجروح. في إطار تحليل حساسية مونت كارلو حساسية المجموعة الثانية كانت فعالة من حيث التكلفة في 80٪ من الحالات بدءا من الاستعداد للدفع من 80 سنت لكل يوم خالي من الجروح.

الاستنتاجات: قدمت المجموعة الثانية العلاج الأكثر فعالية من حيث التكلفة. البديل غير العلاج ليس خيارا في إدارة قرح داء الليشمانيات الجلدي المزمنة. يجب اللجوء إلى استخدام الأسلوب المتبع مع المجموعة الثالثة علاج الجروح الرطب كحد أدنى. وتعتمد فعالية التكلفة في المجموعة الثالثة على عدد الضمادات اللازمة حتى إغلاق الجرح الكامل.

Translated from English version into Arabic by Mahmoud Sami, through

TRANSLATORS
WITHOUT BORDERS

阿富汗慢性皮肤利什曼病湿润创面护理法的卫生经济评价

Stahl, Hans-Christian, Ahmadi, Faridullah; Nahzat, Sami Mohammad; Dong, Hengjin; Stahl, Kurt-WilhelmandSauerborn, Rainer

摘要

引言：该卫生经济评估旨在向阿富汗公共卫生决策者和卫生保健管理人员提供有效地资源分配策略，对由热带利什曼原虫或硕大利什曼原虫引起的皮肤利什曼病患者进行适当的治疗。

方法：使用决策树分析阿富汗皮肤利什曼病患者两种创口护理疗法与皮损内锑疗法的成本和效果。从社会角度收集计算成本，效果则用无创口天数来衡量，分别计算增量成本效用比和增量净货币收益。用基线参数、灵敏度范围和参数分布对模型进行参数化。最后对模型进行模拟，并用确定性和概率敏感性分析评估结果。最终的结果是在阿富汗的不同疗法治疗效

率和预算影响分析。
结果: 三组慢性皮肤利什曼病患者平均每人每处溃疡创口花费分别为 11 美元(I 组: IL SSG，*SE*=0.016)、16 美元（II 组: ETC+MWT，*SE*= 7.58）和 25 美元（III 组: MWT，*SE* = 0.48)。与世卫组织东地中海区皮肤利什曼病葡萄糖酸锑钠疗法病例管理方法相比，II 组和 III 组将分别节省 35.3 万美元和 3.6 万美元药费。I 组、II 组和 III 组的效果（无创口天数）分别为

Translated from English version into Chinese by Chui-Zhao Xue, edited by Pin Yang

CHINESE CENTER FOR DISEASE CONTROL AND PREVENTION
NATIONAL INSTITUTE OF PARASITIC DISEASES

Évaluation en économie de la santé des pansements humides dans le traitement des ulcères cutanés chroniques de la leishmaniose en Afghanistan

Stahl, Hans-Christian, Ahmadi, Faridullah, Nahzat, Sami Mohammad, Dong, Hengjin, Stahl, Kurt-Wilhelm et Sauerborn, Rainer

Résumé

Contexte : Les présentes évaluations en économie de la santé, menées en Afghanistan, ont pour but d’aider les décideurs en matière de santé publique et les gestionnaires des soins à affecter efficacement les ressources pour le traitement adéquat de la leishmaniose cutanée causée par *L. tropica* ou *L. major*.

Méthodes : Nous avons utilisé un arbre de décision afin d’analyser le coût et l’efficacité de deux régimes de traitement des plaies par rapport à l’antimoine intra-lésionnel chez des patients atteints de leishmaniose cutanée en Afghanistan. Les coûts ont été relevés du point de vue sociétal. L’efficacité a été mesurée en jours sans plaies. Le rapport coût-efficacité incrémentiel et le bénéfice monétaire net incrémentiel ont été calculés. Le modèle a été paramétré avec les paramètres au début de l’étude, les fourchettes de sensibilité et les distributions des paramètres. Il a ensuite a été simulé et les résultats évalués par des analyses déterministes et de sensibilité des probabilités. Les paramètres d’évaluation finaux étaient l’efficacité des régimes thérapeutiques et l’impact budgétaire dans le contexte afghan.

Résultats

Le coût moyen par patient était de 11 dollars U.S. (*SE* = 0,016) (groupe I : IL SSG), 16 dollars U.S. (*SE* = 7,58) (groupe II : ETC+MWT) et 25 dollars (*SE* = 0,48) (groupe III : MWT) chez des patients présentant un seul ulcère chronique de leishmaniose cutanée. L’impact budgétaire des régimes II et III représente une économie de 353 000 dollars et 36 000 dollars en frais de médicaments, respectivement, par rapport au protocole de gestion des cas IL SSG du BRMO-OMS. L’efficacité moyenne, en jours sans plaie, était de 177 (*SE* = 0,36) dans le groupe II, 147 (*SE* = 0,33) dans le groupe III et 129 (*SE* = 0,27) dans le groupe I. L’ICER du groupe II par rapport au groupe I était de 0,09 dollar U.S. et celui du groupe III par rapport au groupe II de 0,77 dollar U.S., ce qui est très économique alors que le seuil d’acceptation de payer est de 2 dollars U.S. par jour sans plaie. Selon une analyse de sensibilité probabiliste de Monte-Carlo, le groupe II était économique dans 80 % des cas, avec une acceptation de payer commençant à 80 *cents* par jour sans plaie.

Conclusions : Le groupe II a donné le traitement le plus efficace. L’abstention thérapeutique n’est pas une option dans la prise en charge des ulcères chroniques de leishmaniose cutanée. Le protocole de traitement des plaies par pansements humides du groupe III devrait au moins être essayé. La rentabilité de ce groupe dépend du nombre de pansements nécessaires jusqu’à la cicatrisation complète.

Translated from English version into French by Suzanne Assenat, through

TRANSLATORS
WITHOUT BORDERS

Экономическая оценка терапии влажных ран при хронических язвах кожного лейшманиоза в Афганистане

Шталь, Ханс-Кристиан, Ахмади, Фаридуллах; Нахзат, Сами Мохаммад; Донг, Хенгжин; Шталь, Курт-Вильгельм и Сауерборн, Райнер

Аннотация

Данные: Нынешняя оценка экономики здравоохранения в Афганистане направлена на оказание поддержки органам, ответственным за принятие решений в области здравоохранения, и руководителям медико-санитарного звена в целях эффективного распределения ресурсов на соответствующие методы лечения кожного лейшманиоза, вызванного *L. tropicaor L. major*.

Методы: Метод дерева принятия решений был использован для анализа стоимости и эффективности двух режимов лечения травм по сравнению с внутриочаговым применением препарата сурьмы у пациентов в Афганистане. Затраты были подсчитаны и с позиции общества в целом. Эффективность измеряется днями от момента заживления раны. Были рассчитаны коэффициент дополнительной рентабельности и прирост чистой денежной выгоды. Модель была параметризована по исходным параметрам, диапазонами чувствительности и распределением параметров. Наконец, после имитационного моделирования оценили результаты с помощью детерминированного и вероятностного анализов чувствительности. Конечными результатами являются эффективность режимов и анализ последствий для бюджета в контексте Афганистана.

Результаты

Средняя стоимость на пациента составляла US $11 (SE = 0,016) (группа I: внутриочаговое применение стибоглюконата натрия), US$ 16 (*SE* = 7.58) (группа II: электротермокоагуляция+ терапия влажных ран) и 25 US$ (*SE* = 0.48) (группа III: терапия влажных ран) у пациентов с единичной хронической язвой кожного лейшманиоза. Применение режима II и III для бюджета позволили бы сэкономить $353 000 и US $36000 на лекарственные препараты, соответственно, по сравнению с протоколом Регионального бюро для стран Восточного Средиземноморья по внутриочаговому применению стибоглюконата натрия. Средняя эффективность дней от момента заживления раны составляет 177 (SE = 0,36) в группе II, 147 (SE = 0,33) в группе III и 129 (SE = 0,27) в группе I. ICEP группы II по сравнению с группой I было US $0,09 и группа III по сравнению с группой I US $0,77, что является весьма эффективным с точки зрения затрат с лимитом оплаты в $2 на день от момента заживления ран. В вероятностном анализе чувствительности от момента заживления ран в Монте-Карло группа II была эффективна с точки зрения затрат в 80% случаев, начиная с лимита оплаты в 80 центов на день от момента заживления ран.

Выводы: Группа II показала наиболее эффективное с точки зрения затрат лечение. Альтернативный вариант без лечения не является вариантом терапии хронической язвы кожного лейшманиоза. Группа III - терапия влажных ран - по крайней мере, можно применять в практике. Эффективность с точки зрения затрат группы III зависит от количества необходимых перевязок до полного закрытия раны.

Translated from English version into Russian by TatianaKary, through

Evaluacion económica del estado de salud y del cuidado de la humedad en ulceras crónicas de leishmaniasis cutánea en Afganistán

Stahl, Hans-Christian, Ahmadi, Faridullah; Nahzat, Sami Mohammad; Dong, Hengjin; Stahl, Kurt-Wilhelm y Sauerborn, Rainer

Resumen

Contexto: Las evaluaciones economico sanitarias existentes en Afganistan tienen como objetivo respaldar a los encargados de la toma de decisiones dentro del ámbito de la salud pública así como a los directivos de atención médica con el fin de que asignen recursos de manera eficiente durante los tratamientos de Leishmaniasis cutánea provocada por *L. tropica* o *L. major.*

Métodos: Se hizo uso de un árbol de decisiones que permitió analizar el coste y la efectividad de los dos regímenes de cuidados de heridas, frente a la utilización del antimonio intralesional en pacientes con LLC en Afganistán. La recogida de datos sobre los costes se realizó desde una perspectiva social. La efectividad, se midió en "días sin heridas". Se calculó la relación costo-efectividad incremental y el beneficio monetario neto incremental. Se utilizaron parámetros de referencia, rangos de sensibilidad y distribuciones de parámetros. Finalmente, se llevó a cabo la simulación del modelo, se evaluaron los resultados mediante una serie de análisis de sensibilidad determinado así como con análisis de sensibilidad de probabilidad. Los resultados finales fueron: la eficiencia de los regímenes y un análisis de impacto presupuestario en el contexto de Afganistán.

Resultados:

Coste promedio por paciente fue de 11 USD (SE = 0.016) (Grupo I: IL SSG), 16 USD (SE = 7.58) (Grupo II: ETC+MWT) y 25 USD (*SE* = 0.48) (III: MWT) en pacientes con una única úlcera LCC crónica. El impacto presupuestario de los regímenes II y III ahorraría 0,353 USD millones y 0,036 USD millones respectivamente, en costos de medicamentos en comparación con el protocolo de gestión de casos WHO EMRO IL SSG de la OMS. La efectividad media en días sin herida es de 177 (SE = 0,36) en el Grupo II, 147 (SE = 0,33) en el Grupo III y 129 (SE = 0,27) en el Grupo I. La relación de coste-efectividad incremental del Grupo II frente al Grupo I fue de 0,09 USD; el Grupo III fremte al Grupo I 0,77 USD; lo cual es muy rentable con un umbral de disposición de pago de 2 USD por cada día sin herida. Dentro del ámbito de un análisis de sensibilidad de probabilidad de Monte-Carlo, el grupo II fue rentable en el 80% de los casos. Además cuenta con una disposición de pago de 0.80 cent. por cada día sin heridas.

Conclusiones: El grupo II proporcionó el tratamiento más rentable. La alternativa a un posible no-tratamiento no es una opción para la gestión de úlceras LCC crónicas. MTW del grupo III debería al mensos se reconsiderda como una posibilidad a practicar. La rentabilidad de este último grupo (Grupo III) depende de la cantidad de apósitos necesarios hasta el cierre completo de la herida.

Translated from English version into Spanish by Tamara_jj, through
