# Supplementary material for: Health economic evaluation of moist wound care in chronic cutaneous leishmaniasis ulcers in Afghanistan
Source: Infect Dis Poverty. 2018 Feb 14;7:12. doi: 10.1186/s40249-018-0389-4 (PMC5812215; doi:10.1186/s40249-018-0389-4)

Additional file 2: Decision analytical model. Three treatment alternatives in patients with open chronic cutaneous Leishmaniasis wounds.

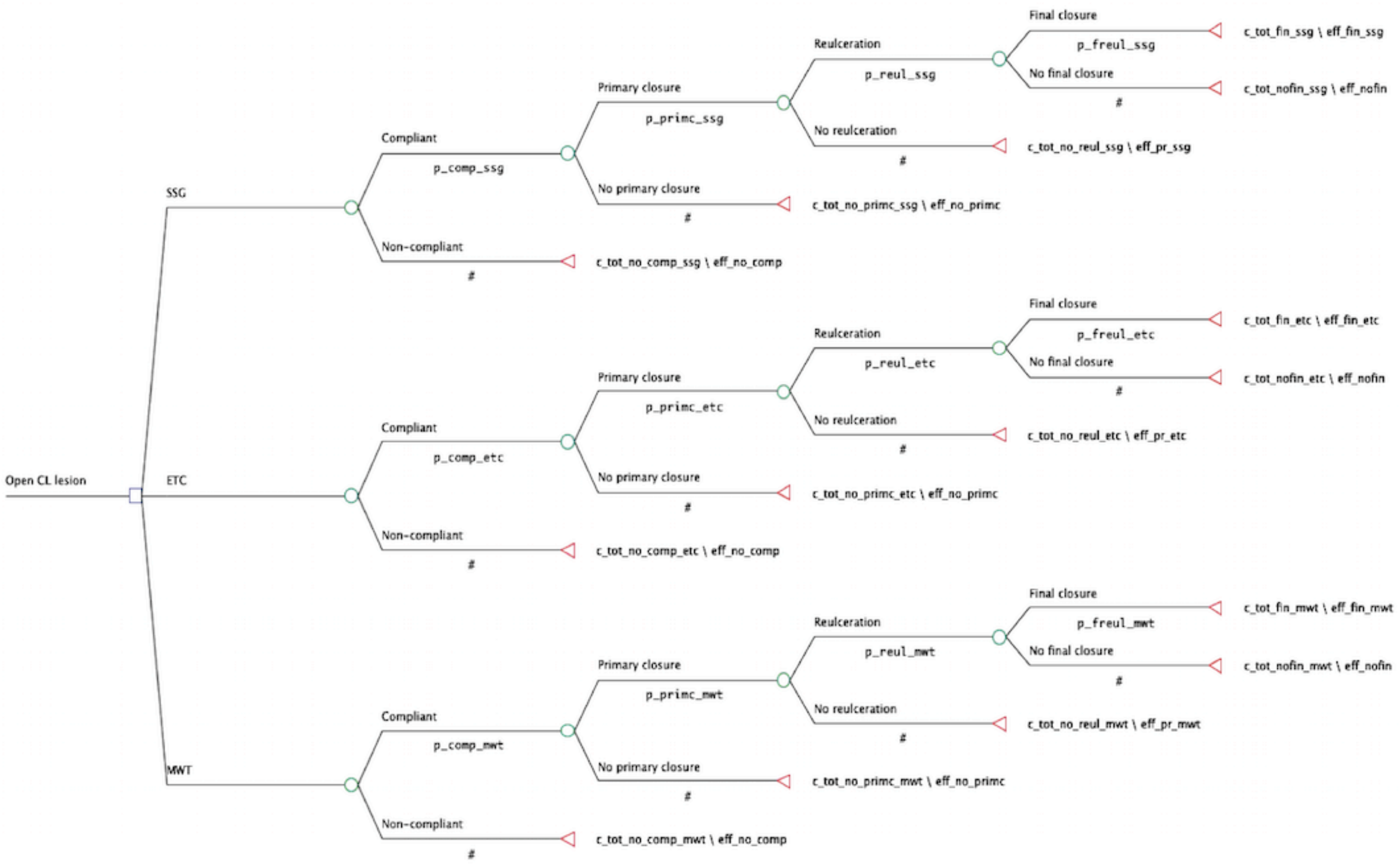

Supplement: Supplementary file 2 — Decision analytical model. Three treatment alternatives in patients with open chronic cutaneous Leishmaniasis wounds. (PDF 281 kb) [file 40249_2018_389_MOESM2_ESM.pdf]
