# Supplementary material for: Health economic evaluation of moist wound care in chronic cutaneous leishmaniasis ulcers in Afghanistan
Source: Infect Dis Poverty. 2018 Feb 14;7:12. doi: 10.1186/s40249-018-0389-4 (PMC5812215; doi:10.1186/s40249-018-0389-4)
Supplement: Supplementary file 3 — Calibration of the decision tree with model assumptions and decision nodes probabilities, Efficacy parameters, Direct medical costs, Direct non-medical costs, Indirect costs. Baseline values, sensitivity ranges and distributions. (PDF 287 kb) [file 40249_2018_389_MOESM3_ESM.pdf]

Table S1: Calibration of the decision tree with model assumptions and decision nodes probabilities. Baseline values, sensitivity ranges and distributions. *Triangular (min, max, modus), *Beta (alpha, beta)

Parameter	Baseline value	Sensitivity From	Sensitivity To	Distribution	Source
**Assumptions**					
Number of lesion	1	1	4	None	Model
Duration of disease	303	180	365	Triangular (180, 365, 365)*	Muray et al. (2005)
Non-compliant cost rate	0.25	0	0.5	Triangular (0, 0.5, 0.25)	Educated guess
Per capita income	700	700	1000	None	World bank
**Node probabilities SSG**					
Treatment compliance	0.614	0.5526	0.6754	Beta (148, 93)*	Reithinger et al. (2015)
Primary closure	0.957	0.8613	1	Beta (22, 1)	Stahl et al. (2014)
Reulceration	0.182	0.1638	0.2000	Beta (4, 18)	Stahl et al. (2014)
Final closure after reulceration	0.75	0.6750	0.8250	Beta (3, 1)	Stahl et al. (2014)
**Node probabilities ETC**					
Treatment compliance	0.719	0.6471	0.7909	Beta (23, 9)	Stahl et al. (2014)
Primary closure	1	0.9000	1	Beta (22.75, 0.25)	Stahl et al. (2014)
Reulceration	0.13	0.1170	0.1430	Beta (3, 20)	Stahl et al. (2014)
Final closure after reulceration	0.667	0.6003	0.7337	Beta (2, 1)	Stahl et al. (2014)
**Node probabilities MWT**					
Treatment compliance	0.742	0.6678	0.8162	Beta (23, 8)	Stahl et al. (2014)
Primary closure	1	0.9000	1	Beta (22.75, 0.25)	Stahl et al. (2014)
Reulceration	0.304	0.2736	0.3344	Beta (7, 16)	Stahl et al. (2014)
Final closure after reulceration	0.286	0.2574	0.3146	Beta (2, 5)	Stahl et al. (2014)

Table S2: Efficacy parameters. Baseline values, sensitivity ranges and distributions. *Triangular (min, max, modus)

Parameter	Baseline value	Sensitivity		Distribution	Source
		From	To		
**Efficacy SSG**					
Mean days for primary closure	69	49	89	Triangular (49, 89, 69)*	Stahl et al. (2014)
Mean days of reulceration	15	7	27	Triangular (7, 27, 11)	Stahl et al. (2014)
Mean days between primary closure and reulceration	27	11	44	Triangular (11, 44, 26)	Stahl et al. (2014)
Mean days for final closure	96	57	136	Triangular (57, 136, 95)	Stahl et al. (2014)
**Efficacy ETC**					
Mean days for primary closure	33	29	37	Triangular (29, 37, 33)	Stahl et al. (2014)
Mean days of reulceration	32	16	48	Triangular (16, 48, 32)	Stahl et al. (2014)
Mean days between primary closure and reulceration	53	25	107	Triangular (25, 107, 27)	Stahl et al. (2014)
Mean days for final closure	136	116	156	Triangular (116, 156, 136)	Stahl et al. (2014)
**Efficacy MWT**					
Mean days for primary closure	45	34	56	Triangular (34, 56, 45)	Stahl et al. (2014)
Mean days of reulceration	41	14	67	Triangular (14, 67, 41)	Stahl et al. (2014)
Mean days between primary closure and reulceration	39	12	66	Triangular (12, 66, 39)	Stahl et al. (2014)
Mean days for final closure	71	42	99	Triangular (42, 99, 71)	Stahl et al. (2014)

Table S3: Direct medical costs. Baseline values, sensitivity ranges and distributions. *Gamma (apha, beta)

Parameter	Baseline value	Sensitivity From	Sensitivity To	Distribution	Source
**Direct medical cost SSG**					
**Drug SSG**					
Cost per ml SSG	0.22	0.194	1	Gamma (100, 454)*	Albert David, India
Dosage of SSG per wound (ml)	0.60	0.540	0.660	Normal (0.6, 0.06)	Stahl et al. (2014)
**Desinfection**					
Cost per ml ethanol	0.004	0.0036	0.0044	Gamma (100, 25000)	Pharmacy MeS
Ethanol dosage per wound (ml)	2	1	3	Normal (2, 1)	Stahl et al. (2014)
**Disposables**					
Cost syringe (1cc) per unit	0.06	0.054	0.066	Gamma (100, 1666)	Pharmacy MeS
Cost gloves per pair	0.07	0.063	0.077	Gamma (100, 1428)	Pharmacy MeS
Cost gauze per unit	0.02	0.018	0.022	Gamma (100, 5000)	Pharmacy MeS
**Number of injection**					
Number of injection for primary closure	12	10	14	None	Stahl et al. (2014)
Average days between two injections	3	2	4	Normal (3, 1)	Stahl et al. (2014)

Table S3 (continued): Direct medical costs. Baseline values, sensitivity ranges and distributions. *Gamma (apha, beta)

Parameter	Baseline value	Sensitivity		Distribution	Source
		From	To		
**Direct medical cost ETC**					
**Fix costs: ETC**					
**ETC Medical device**					
Unit price ETC Minicutter	2040.97	1.836.873	4.080	Gamma (100, 0.048)	KLS Martin
Lifetime ETC device	10	5	20	Triangular (5, 20, 5)	KLS Martin
Wounds per year treated with ETC	2000	500	4000	Triangular (500, 4000, 1500)	Model
**Anesthesia of the lesion**					
Lidocain 1% injection solution per ml	0.03	0.027	0.033	Gamma (100, 3333)	Pharmacy in MeS
Dosage lidocain (ml) per wound	2	1	3	Normal (2,1)	Stahl et al. (2014)
**Syringe for basic creme**					
Syringe for basic creme (20cc)	0.04	0.036	0.044	Gamma (100, 2500)	Pharmacy in MeS
**Variable costs: Dressing costs**					
**Jelly or Basic creme DAC N-055**					
Cost per g of DAC N-055 Basic creme or jelly	0.085	0.085	1	Gamma (100, 1176)	Waisenmedizin e.V.
Dosage of DAC N-055 Basic creme or jelly per wound (g)	2	1	3	Normal (2,1)	Stahl et al. (2014)
**Desinfection**					
Cost per ml ethanol	0.04	0.036	0.044	Gamma (100, 25000)	Pharmacy in MeS
Dosage per wound ethanol (ml)	2	1	3	Normal (2, 1)	Stahl et al. (2014)
**Disposables**					
Cost gauze per unit	0.02	0.018	0.022	Gamma (100, 5000)	Pharmacy in MeS
Cost gloves per pair	0.07	0.063	0.077	Gamma (100, 1428)	Pharmacy in MeS
Cost Leukoplast (30cm)	0.01	0.009	0.011	Gamma (100, 10000)	Pharmacy in MeS
**Number of dressings**					
Average days between two dressings	3	2	4	Normal (3, 1)	Stahl et al. (2014)

Table S3 (continued): Direct medical costs. Baseline values, sensitivity ranges and distributions. *Gamma (apha, beta)

Parameter	Baseline value	Sensitivity		Distribution	Source
		From	To		
**Direct medical cost MWT**					
**Fix costs: MWT**					
**Syringe for basic creme**					
Syringe for basic creme (20cc)	0.04	0.036	0.044	Gamma (100, 2500)	Pharmacy in MeS
**Variable costs: Dressing costs**					
**Jelly or Basic creme DAC N-055**					
Cost per g of DAC N-055 Basic creme or jelly	0.085	0.085	1	Gamma (100, 1176)	Waisenmedizin e.V.
Dosage of DAC N-055 Basic creme or jelly per wound (g)	2	1	3	Normal (2, 1)	Stahl et al. (2014)
**Desinfection**					
Cost per ml ethanol	0.004	0.0036	0.0044	Gamma (100, 25000)	Pharmacy in MeS
Dosage per wound ethanol (ml)	2	1	3	Normal (2, 1)	Stahl et al. (2014)
**Disposables**					
Cost gauze per unit	0.02	0.018	0.022	Gamma (100, 5000)	Pharmacy in MeS
Cost gloves per pair	0.07	0.063	0.077	Gamma (100, 1428)	Pharmacy in MeS
Cost Leukoplast (30cm)	0.01	0.009	0.011	Gamma (100, 10000)	Pharmacy in MeS
**Number of dressings**					
Average days between two dressings	3	2	4	Normal (3, 1)	Stahl et al. (2014)

Table S4: Direct non-medical costs. Baseline values, sensitivity ranges and distributions. *Gamma (apha, beta), *Normal (mean, sd)

Parameter	Baseline value	Sensitivity		Distribution	Source
		From	To		
**Direct non-medical cost SSG**					
**Number of visits**					
Mean number of follow-up visits	5	4	6	Normal (5, 1)	Stahl et al. (2014)
**Transportation Cost**					
Mean transportation time per visit (in minutes)	21	18	24	Normal (21, 7)	Stahl et al. (2014)
Mean transportation cost per visit	0.4	0.31	0.49	Gamma (49, 19)	Stahl et al. (2014)
Mean waiting time per visit before treatment (in minutes)	7	6	8	Normal (7, 2)	Stahl et al. (2014)
Mean treatment time per visit (in minutes)	12	11	13	Normal (12, 3)	Stahl et al. (2014)
**Direct non-medical cost ETC**					
**Number of visits**					
Mean number of follow-up visits	4	3	5	Normal (4, 1)	Stahl et al. (2014)
**Transportation Cost**					
Mean transportation time per visit (in minutes)	20	17	23	Normal (21, 7)	Stahl et al. (2014)
Mean transportation cost per visit	0.63	0.43	0.83	Gamma (15, 10)	Stahl et al. (2014)
Mean waiting time per visit before treatment (in minutes)	7	6	8	Normal (7, 3)	Stahl et al. (2014)
Mean treatment time per visit (in minutes)	13	12	14	Normal (13, 3)	Stahl et al. (2014)

Table S4 (continued): Direct non-medical costs. Baseline values, sensitivity ranges and distributions. *Gamma (apha, beta), *Normal (mean, sd)

Parameter	Baseline value	Sensitivity		Distribution	Source
		From	To		
**Direct non-medical cost MWT**					
**Number of visits**					
Mean number of follow-up visits	4	3	6	Normal (4, 1)	Stahl et al. (2014)
**Transportation Cost**					
Mean transportation time per visit (in minutes)	21	18	24	Normal (21, 7)	Stahl et al. (2014)
Mean transportation cost per visit	0.54	0.33	0.75	Gamma (7, 12)	Stahl et al. (2014)
Mean waiting time per visit before treatment (in minutes)	7	6	8	Normal (7, 2)	Stahl et al. (2014)
Mean treatment time per visit (in minutes)	13	12	14	Normal (13, 3)	Stahl et al. (2014)

Table S5: Indirect costs. Baseline values and sensitivity ranges.

Parameter	Baseline value	Sensitivity		Distribution	Source
		From	To		
**Indirect cost SSG**					
**Productivity loss**					
Productivity loss	0.01	0.01	0.2	None	Educated guess
**Indirect cost ETC**					
**Productivity loss**					
Productivity loss	0.01	0.01	0.2	None	Educated guess
**Indirect cost MWT**					
**Productivity loss**					
Productivity loss	0.01	0.01	0.2	None	Educated guess
