# Supplementary figures and images for: Health economic evaluation of moist wound care in chronic cutaneous leishmaniasis ulcers in Afghanistan
Source: Infect Dis Poverty. 2018 Feb 14;7:12. doi: 10.1186/s40249-018-0389-4 (PMC5812215; doi:10.1186/s40249-018-0389-4)

Figure S1

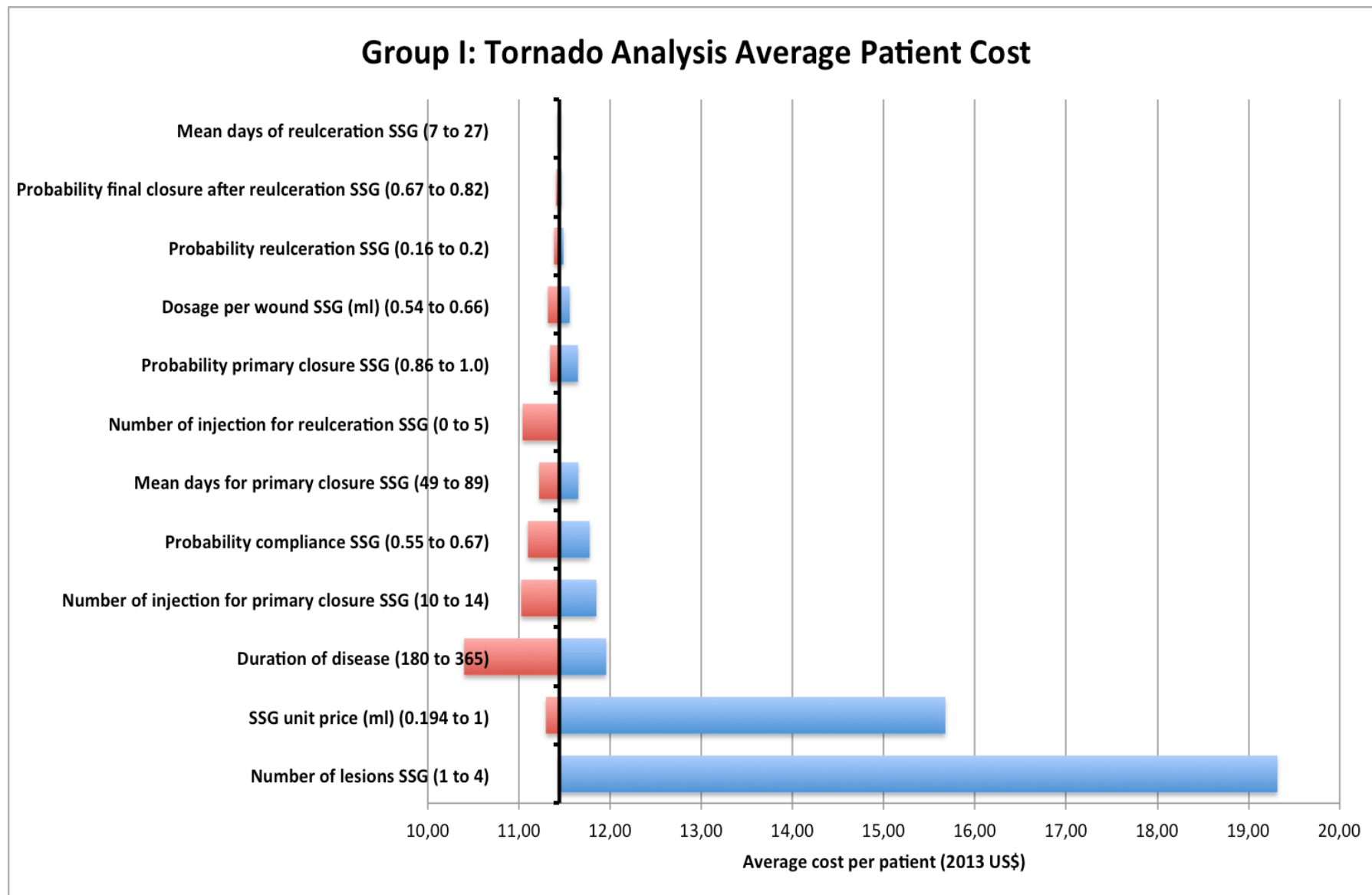

Figure S2

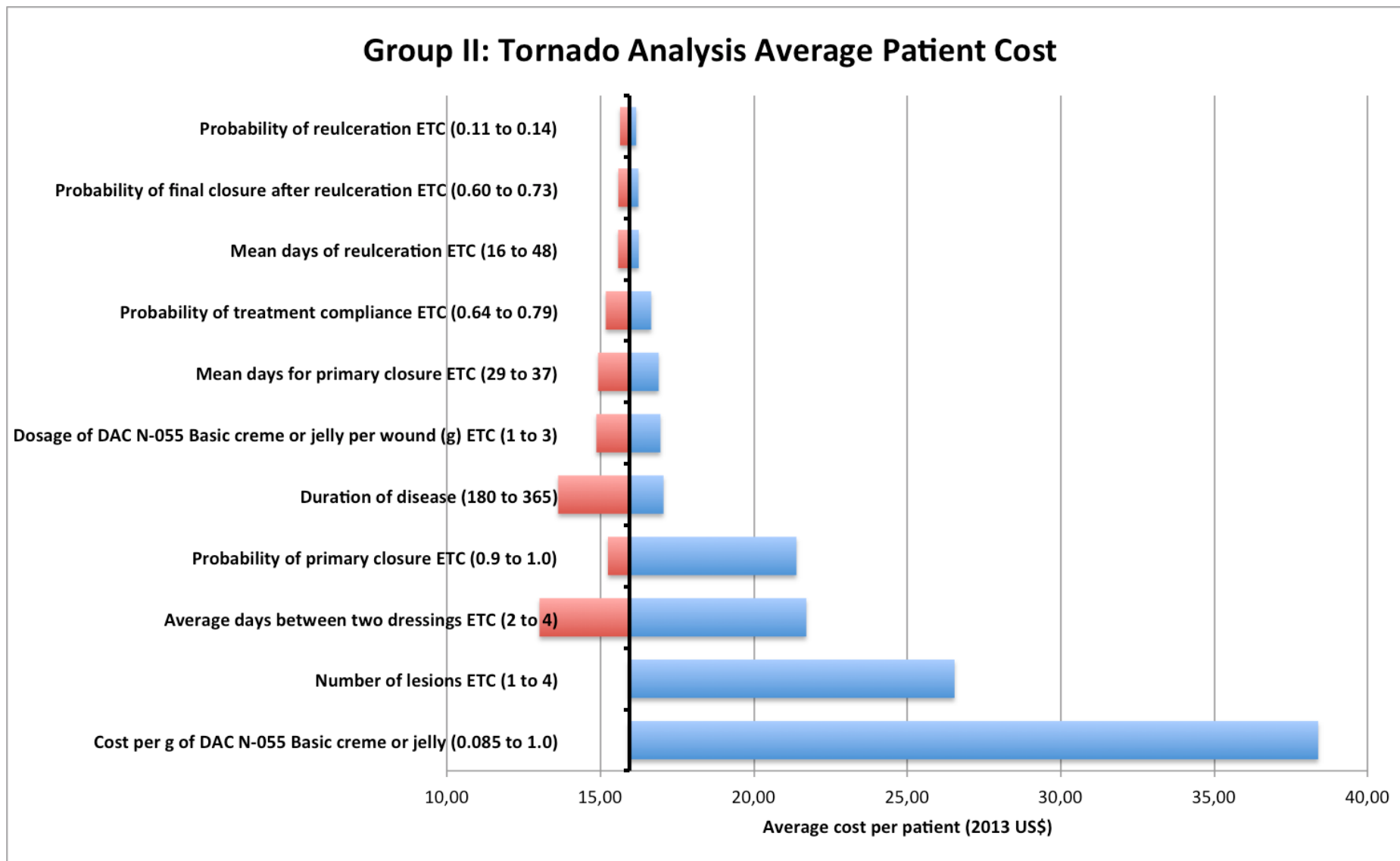

Figure S3

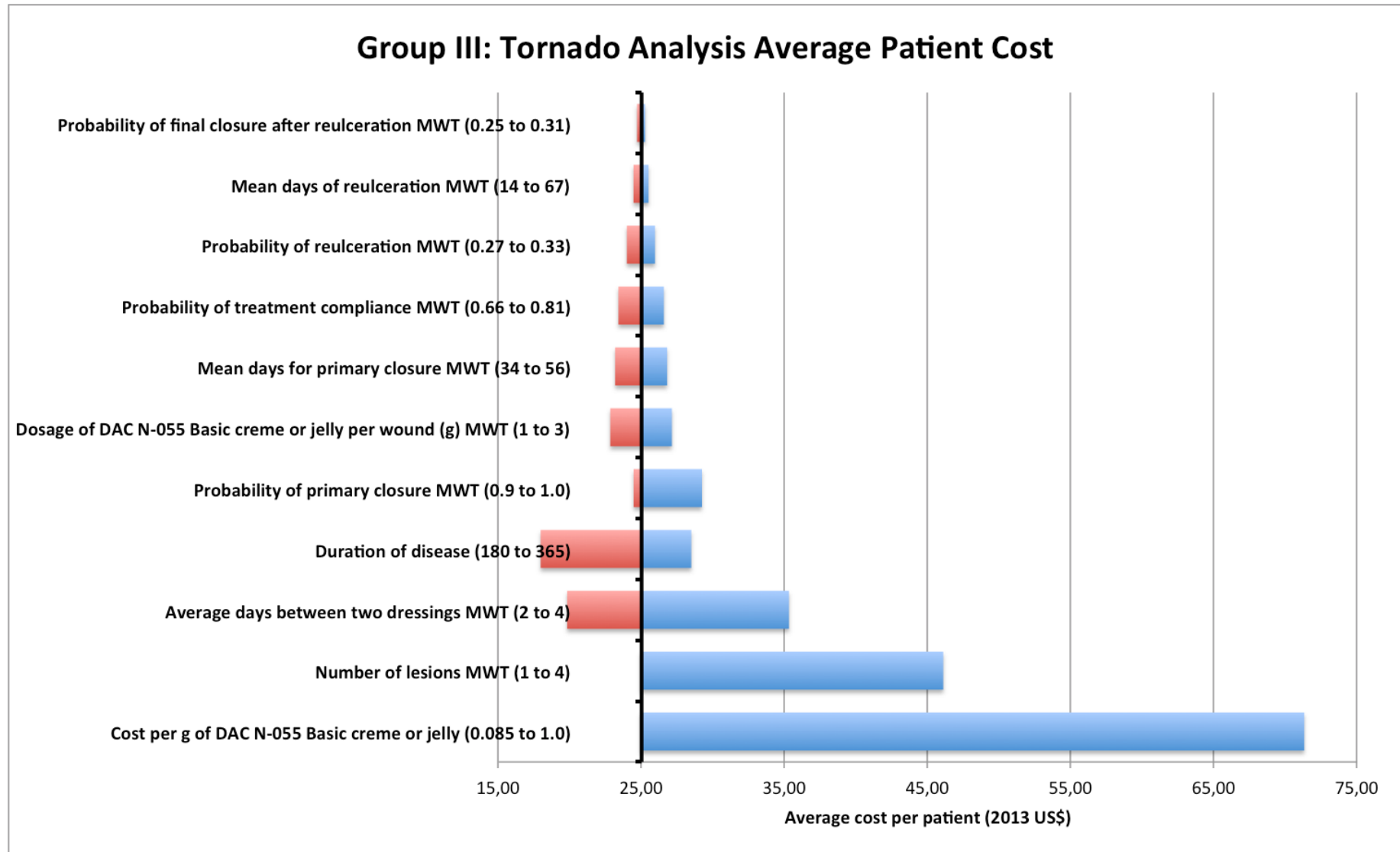

Figure S4

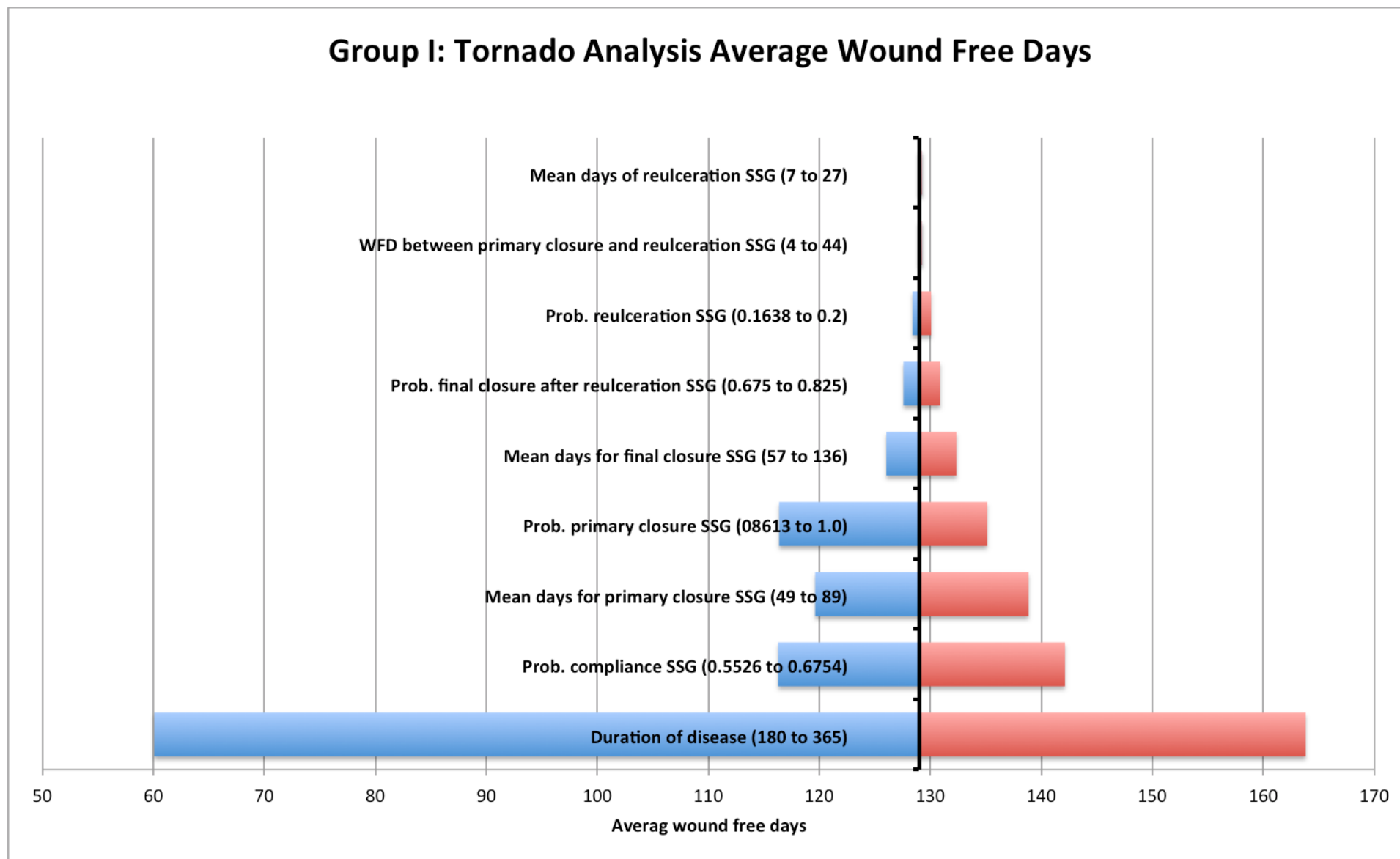

Figure S5

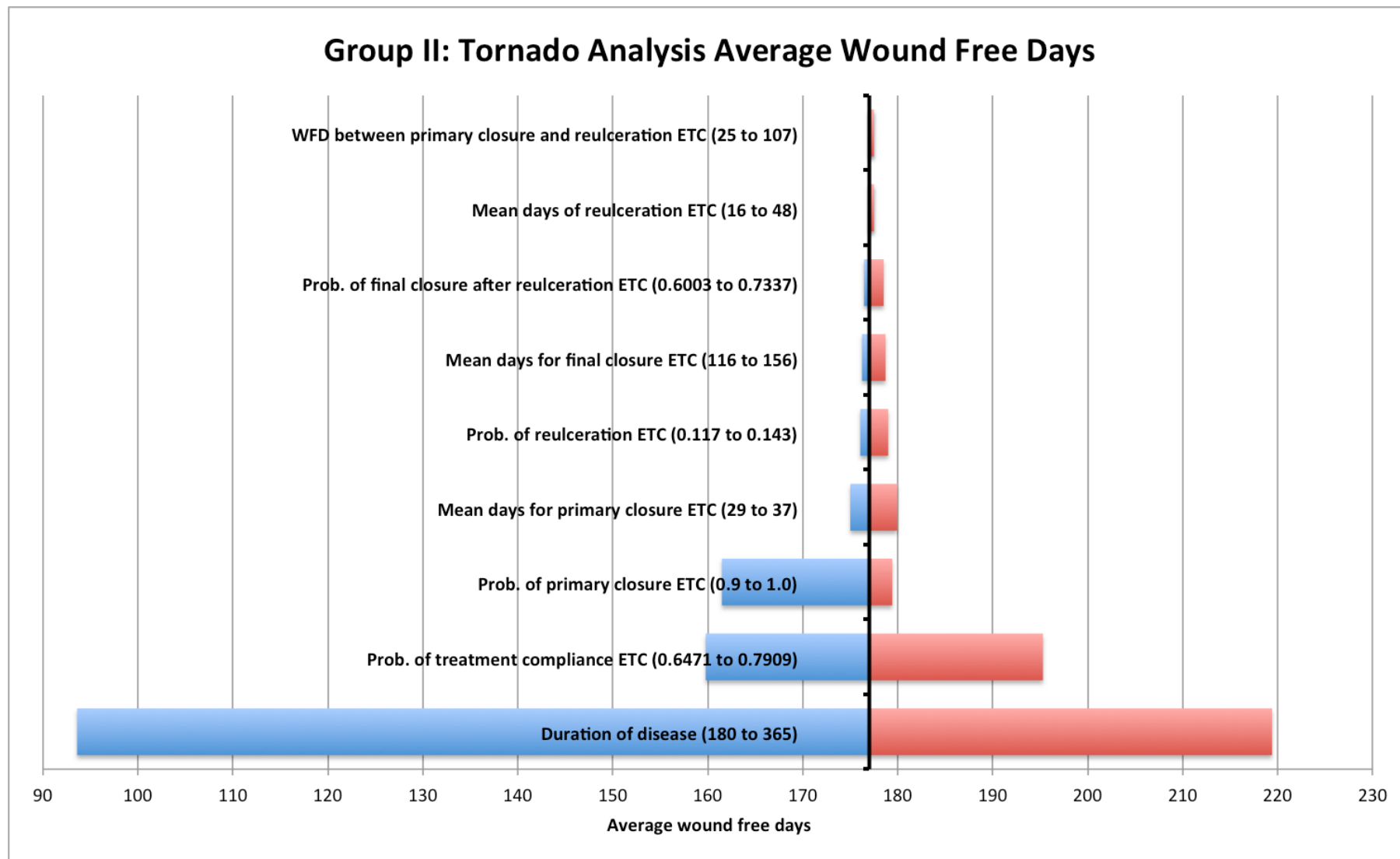

Figure S6

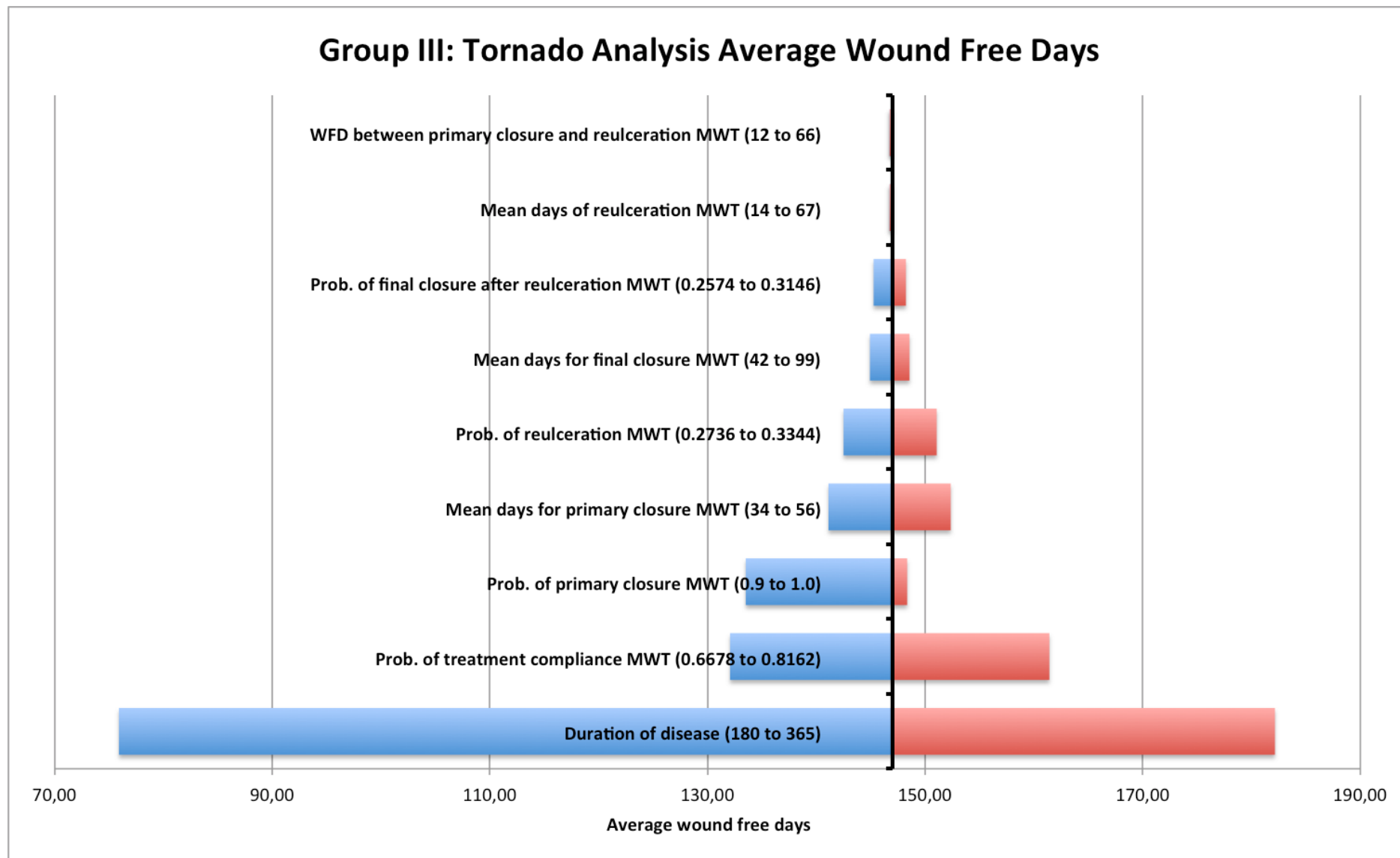

Figure S7

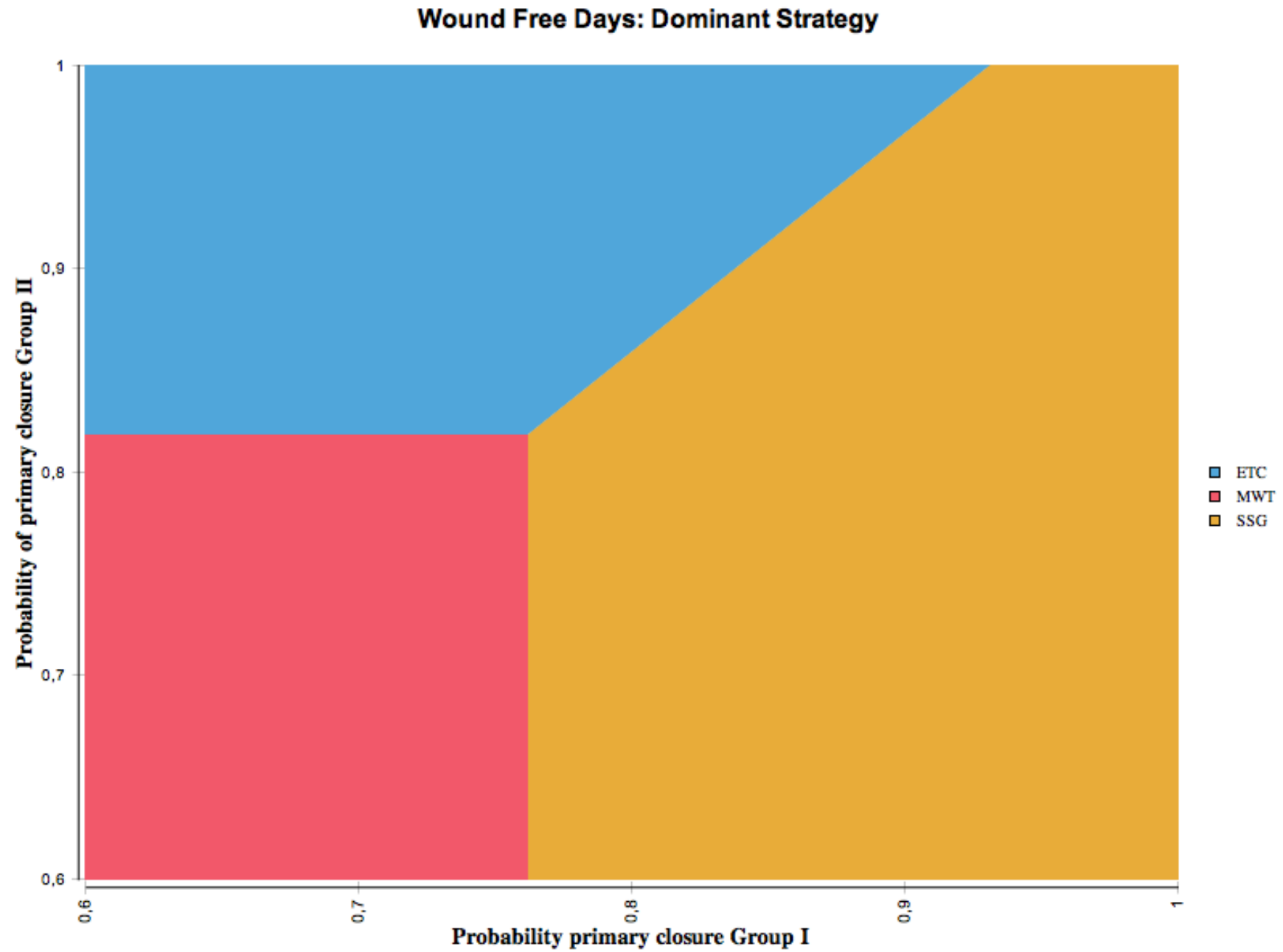

Figure S8

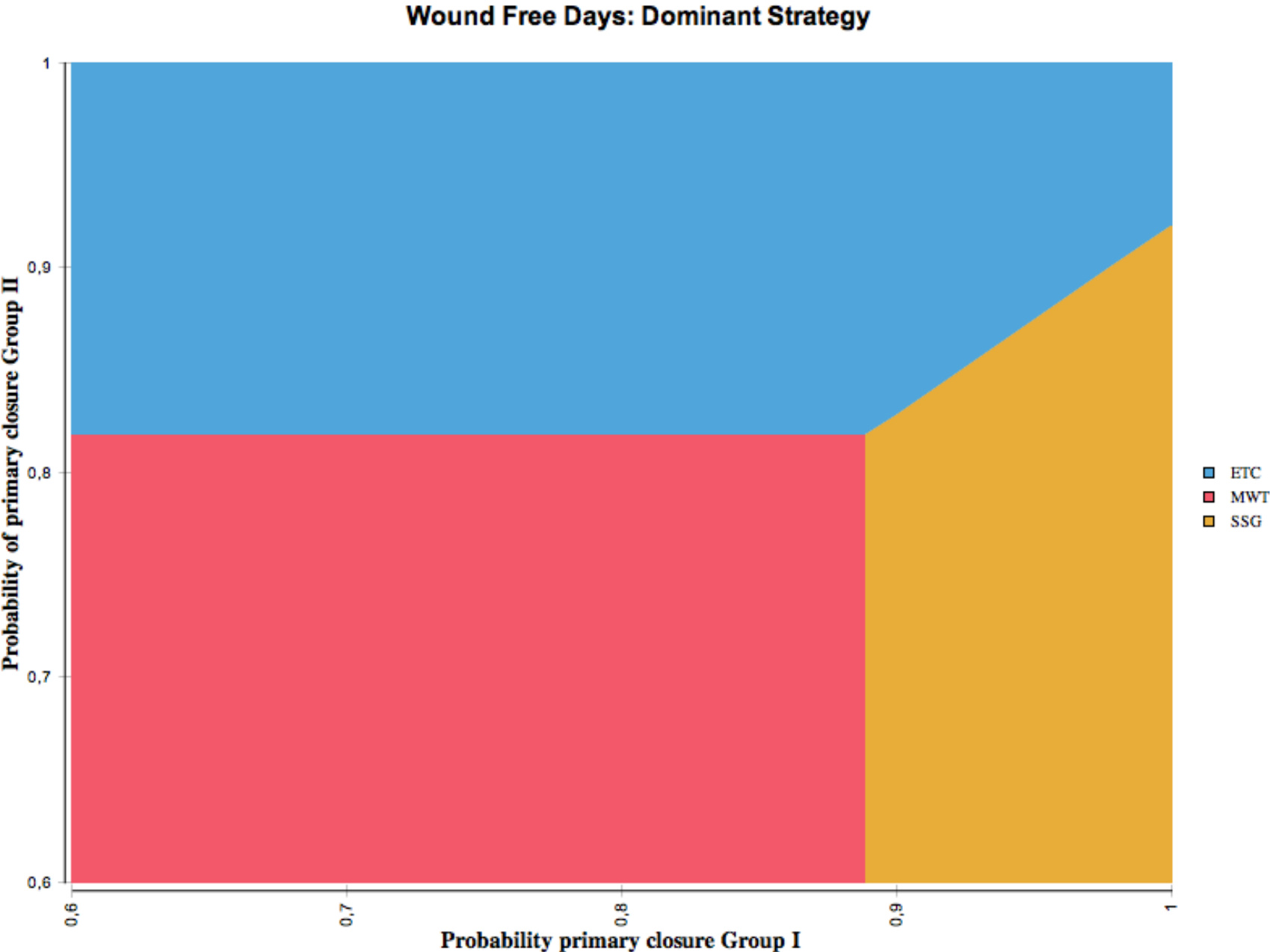

Figure S9

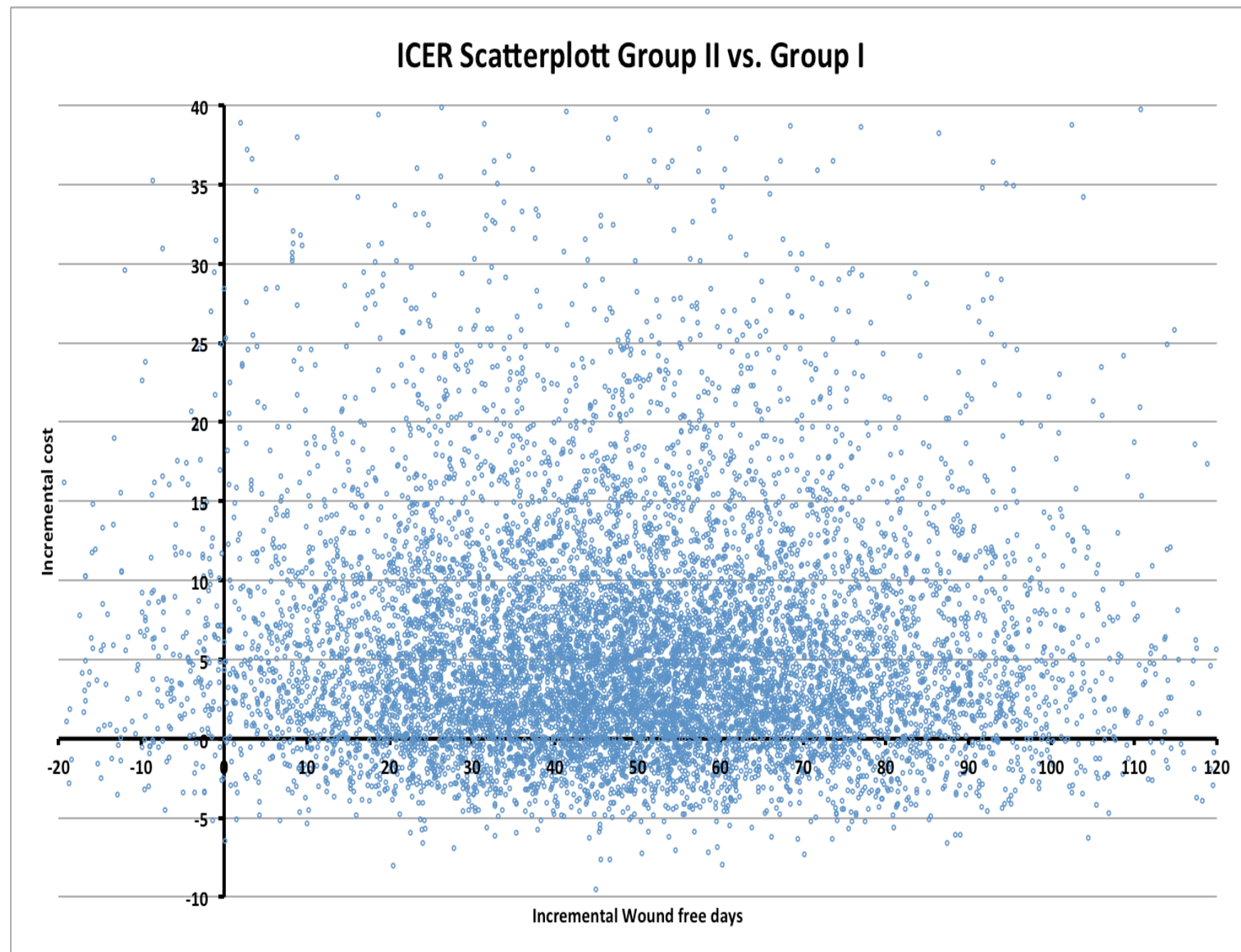

Figure S10

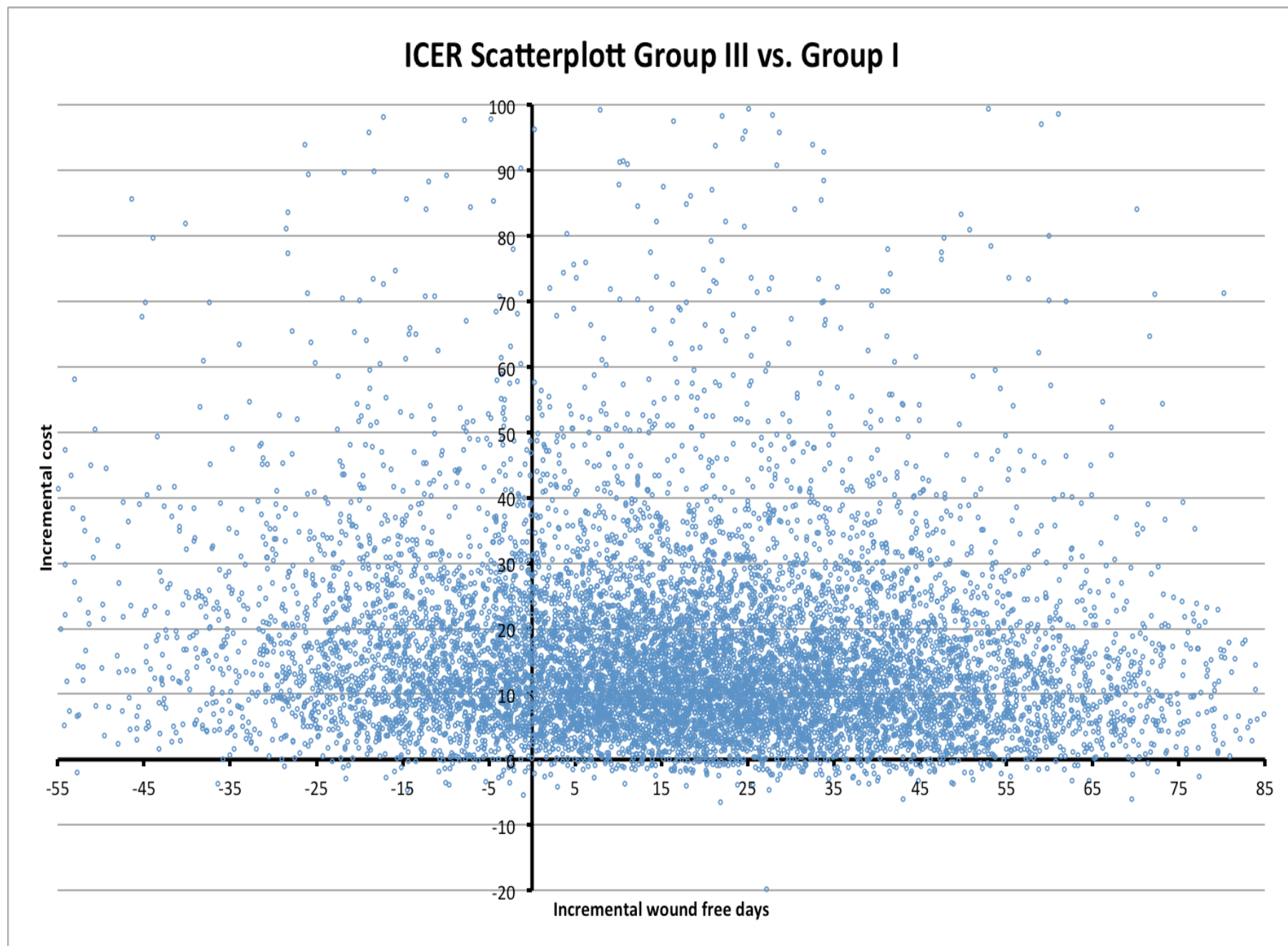

Figure S11

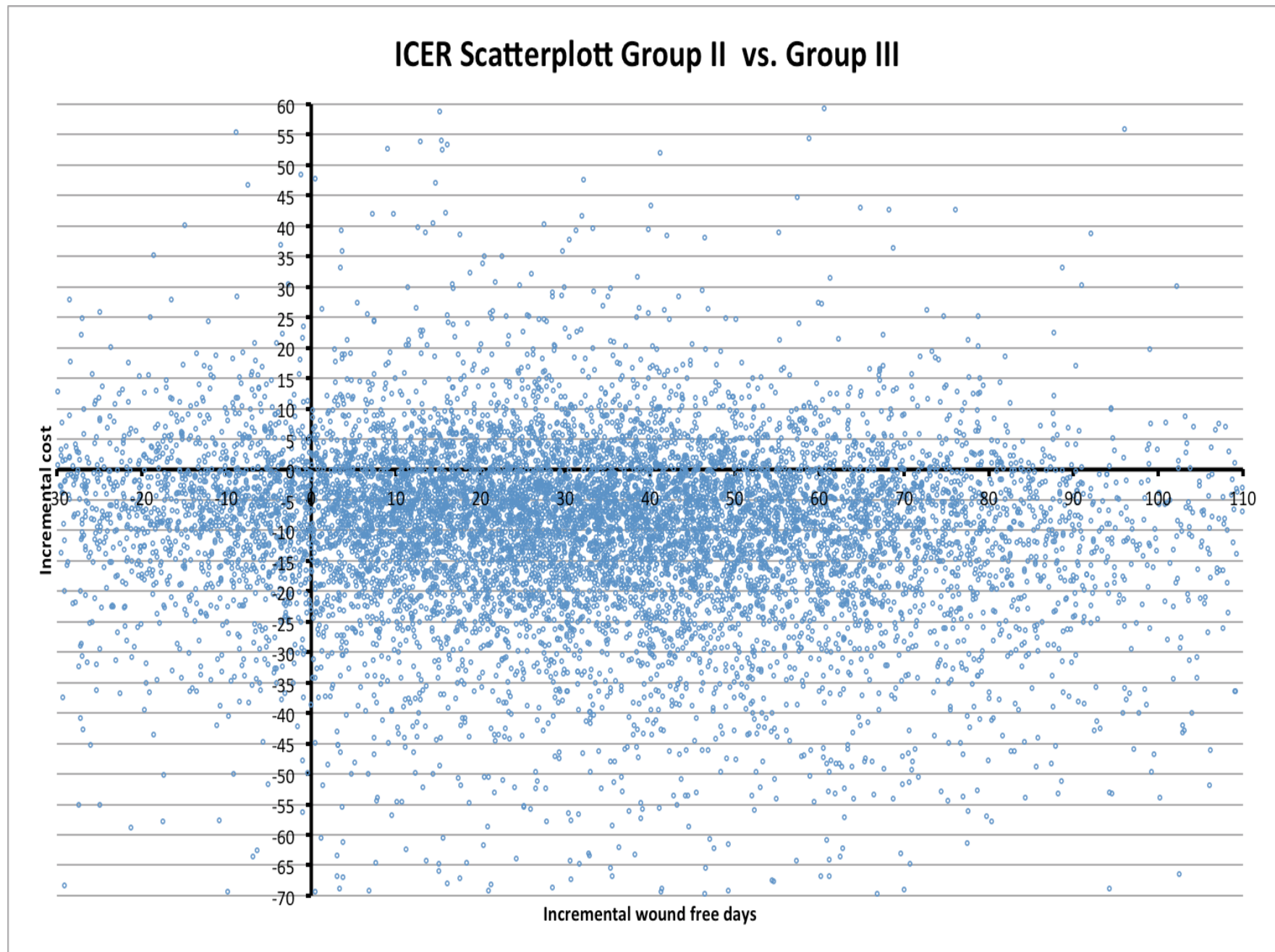

Supplement: Supplementary file 4 — Tornado diagrams, 3-way sensitivity analyses and Cost-Effectiveness Planes. In the 3-way Sensitivity Analysis of Effectiveness represent variation of primary closure probabilities in Group I versus II with a 12.5% (Figure S7) and 25% (Figure S8) non-compliant patients rate in Group I. (PDF 1082 kb) [file 40249_2018_389_MOESM4_ESM.pdf]
